# Phosphorylation of the BNIP3 C-Terminus Inhibits Mitochondrial Damage and Cell Death without Blocking Autophagy

**DOI:** 10.1371/journal.pone.0129667

**Published:** 2015-06-23

**Authors:** Katherine E. Liu, William A. Frazier

**Affiliations:** Department of Biochemistry and Molecular Biophysics, Washington University School of Medicine, St. Louis, Missouri, United States of America; University of Alabama at Birmingham, UNITED STATES

## Abstract

BNIP3 is a dual function protein, able to activate autophagy and induce cell death. Upon expression of BNIP3, which is upregulated by hypoxia, the protein induces mitochondrial dysfunction, often leading to cell death. However, some highly respiring cells and cancer cells tolerate BNIP3 expression, suggesting that a yet unknown mechanism exists to restrain the lethal effects of BNIP3 on mitochondria. Here we present evidence that BNIP3 undergoes several phosphorylation events at its C-terminus, adjacent to the transmembrane domain. Phosphorylation at these residues inhibits BNIP3-induced mitochondrial damage, preventing a loss of mitochondrial mass and mitochondrial membrane potential, as well as preventing an increase in reactive oxygen species. This decrease in mitochondrial damage, as well as the reduction of cell death upon C-terminal BNIP3 phosphorylation, can be explained by a diminished interaction between BNIP3 and OPA1, a key regulator of mitochondrial fusion and mitochondrial inner membrane structure. Importantly, phosphorylation of these C-terminal BNIP3 residues blocks cell death without preventing autophagy, providing evidence that the two functional roles of BNIP3 can be regulated independently. These findings establish phosphorylation as a switch to determine the pro-survival and pro-death effects of the protein. Our findings also suggest a novel target for the regulation of these activities in transformed cells where BNIP3 is often highly expressed.

## Introduction

BNIP3 (BCL2/adenovirus E1B 19 kDa protein-interacting protein 3) expression is transcriptionally upregulated by HIF-1α in hypoxic conditions [1]. Upon expression, BNIP3 localizes to mitochondria, where it collapses mitochondrial membrane potential (ΔΨm), increases generation of reactive oxygen species (ROS), induces mitochondrial swelling, promotes mitochondrial fission, and stimulates mitochondrial turnover via autophagy (mitophagy) [2–6]. Furthermore, when the damaging effects of BNIP3 exceed the ability of the cell to efficiently dispose of damaged mitochondria via mitophagy, programmed cell death can ensue [7, 8]. Each of these effects, including BNIP3-induced mitochondrial damage, stimulation of autophagy, and activation of cell death, require the C-terminal transmembrane (TM) domain of BNIP3 [6, 9].

Evidence suggests that the mitophagy-inducing and the cell death-inducing activities of BNIP3 can be independently regulated [10]. To stimulate activation of mitophagy, BNIP3 functions as a tether, linking BNIP3 localized on damaged mitochondria to LC3-II (microtubule-associated protein 1A/1B-light chain 3) present on nascent autophagosomes [11]. It has been reported that phosphorylation of BNIP3 at S17 and S24, which flank the LC3-II interacting region (LIR, WVEL sequence at residues 18–21), promotes mitophagy through enhanced BNIP3-LC3-II interaction [12]. BNIP3 is also known to increase the localization of DRP1 (Dynamin-related protein 1), a mitochondrial fission protein, to mitochondria, where it stimulates fragmentation of the mitochondrial network to promote the engulfment of damaged mitochondria [13]. This suggests a mechanism by which BNIP3 promotes the selective mitophagy of small, depolarized mitochondria first by acting as a signal for DRP1 to fragment damaged mitochondria, and second by tethering BNIP3-tagged mitochondria to LC3-II-decorated autophagosomes [14].

In addition to the recruitment of DRP1 to the outer mitochondrial membrane to promote mitochondrial fission, BNIP3 has been shown to interact in the mitochondrial intermembrane space with OPA1 (Optic Atrophy 1 (Autosomal Dominant)), a mitochondrial fusion protein localized to the inner mitochondrial membrane [15, 16]. The BNIP3-OPA1 interaction, which inhibits mitochondrial fusion, occurs in the mitochondrial intermembrane space, and is dependent on both the BNIP3 TM domain (residues 164–184) and the ten C-terminal residues distal to the TM domain (residues 185–194) [16]. OPA1 oligomers are also involved in the storage of cytochrome *c*, which is sequestered in pockets formed by the cristae junctions of the inner mitochondrial membrane [17–19]. BNIP3 has been shown to disrupt these OPA1 oligomers, causing cytochrome *c* release and activation of classical apoptosis [15, 16, 20]. However, BNIP3 induces cell death through several pathways, depending on the cell type and physiological conditions [21]. In some cells, BNIP3-induces classical apoptosis, exhibiting characteristic features including release of cytochrome *c* and caspase activation [22, 23]. In other cases, cells die via autophagic cell death or programmed cell death type III, a caspase-independent cell death mechanism characterized by discharge of ΔΨm, loss of ATP generating capacity, externalization of phosphatidylserine, and eventual permeabilization of the cell [24–26].

The dual role of BNIP3 in activating autophagy and/or cell death in the context of transformed cells also appears to be dependent on cell type [27]. For example, BNIP3-induced activation of autophagy has been described as a mechanism used by transformed cells, including colon carcinoma and breast cancer cells, to promote cell survival [28], whereas in some breast and glioma cancer cell lines, BNIP3 promotes autophagic cell death [26]. Some cancer therapies are now addressing the ways in which the balance between autophagy and cell death can be manipulated to selectively sensitize transformed cells to cell death [29]. Here we present evidence that phosphorylation at the extreme C-terminus of BNIP3 controls the ability of the protein to damage mitochondria and activate cell death without blocking its ability to stimulate autophagy. Thus, phosphorylation acts as a switch to control the pro-survival and pro-death functions of BNIP3.

## Results

### The BNIP3 C-terminus contains multiple phosphorylation sites

**Fig 1:**
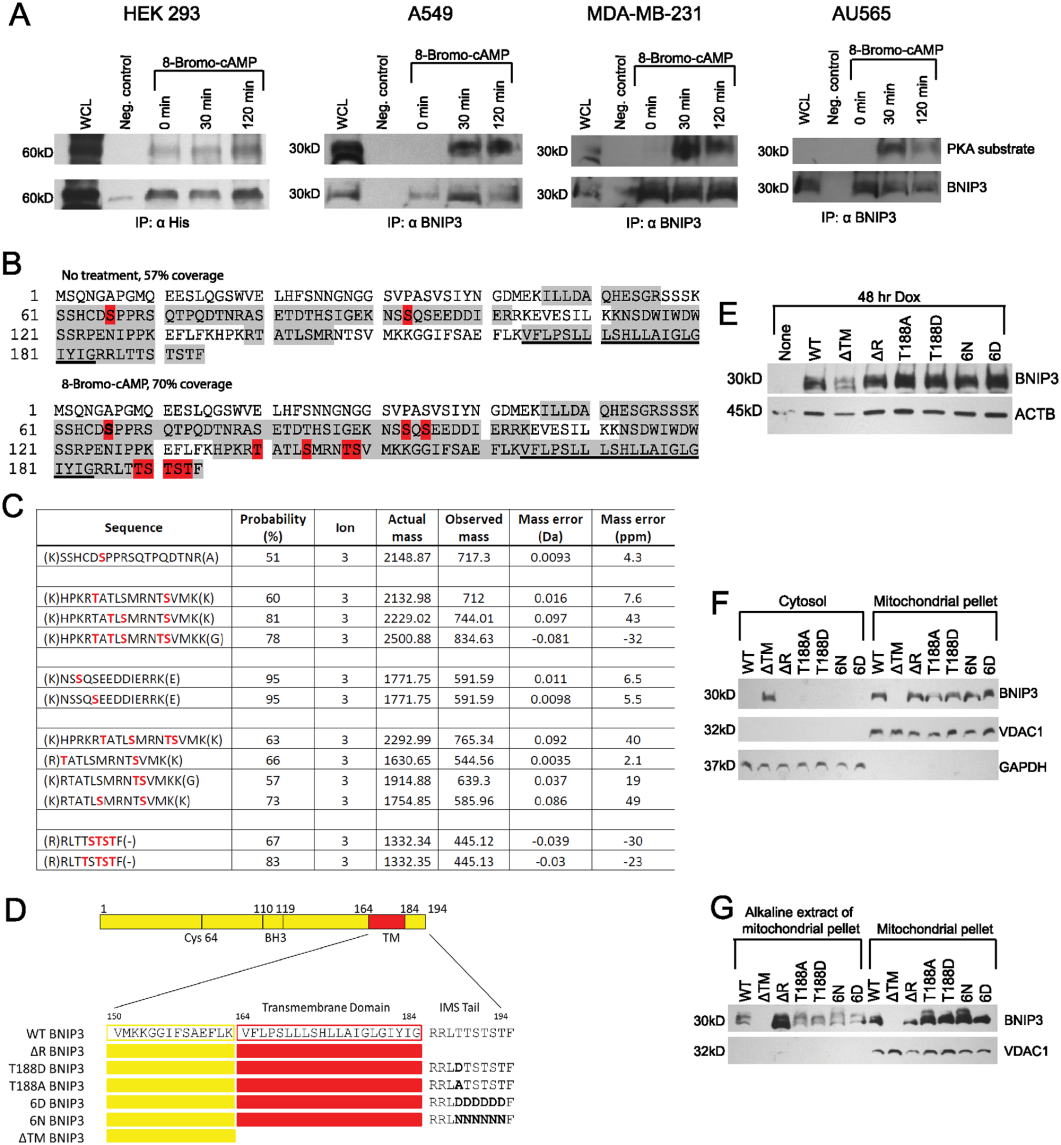
The BNIP3 C-terminus can be phosphorylated. (A) Western blot detection of immunoprecipitated BNIP3 using an α-PKA substrate antibody specific to the phosphorylated RRXS/T sequence, located at the BNIP3 C-terminus and adjacent to the transmembrane (TM) domain. Results shown for 4 cell types: (left to right) HEK 293 cells expressing exogenous BNIP3 (dimer, 60 kD), and A549, MDA-MD-231, and AU565 cells expressing endogenous BNIP3 (monomer, 30kD). Lane 1 of each Western blot contains the whole cell lysate (WCL). (B) LC-MS/MS analysis of BNIP3 phosphorylation in HEK 293 cells with normal or elevated cAMP (8-Bromo-cAMP), showing peptide coverage (gray) and phosphorylation sites (red). The TM domain is underlined. (C) Table of BNIP3 phosphopeptides identified by LC-MS/MS, showing the percent probability, ion charge, actual and observed masses, and mass error (Da and ppm) for each peptide. Peptides shown are from analysis of BNIP3 purified from HEK 293 cells with elevated cAMP levels. (D) Schematic of the BNIP3 protein sequence, showing each C-terminal mutation representing phosphomimetic or nonphosphorylated BNIP3. (E) Expression of BNIP3 phosphomutants from stable doxycycline-inducible HEK 293 Tet On cells, treated with doxycycline (Dox) for 48 hr. (F) Subcellular localization of BNIP3 phosphomutants, showing Western blot of cytosolic and mitochondrial fractions. (G) Alkaline extraction of mitochondria-associated proteins, showing alkaline extract of the mitochondrial pellet and the alkaline-resistant mitochondrial pellet from cells expressing each BNIP3 phosphomutant. All blots are representative of at least 3 independent experiments.

Although BNIP3 is known to be phosphorylated, little is understood about how specific phosphorylation events impact the death-inducing function of BNIP3 [5, 12, 30, 31]. However, it is well established that the BNIP3 TM domain is necessary for BNIP3 function [32]. Interestingly, a series of six S/T residues, including a canonical protein kinase A (PKA) substrate recognition sequence (RRLT, residues 185–188), are located immediately C-terminal to the BNIP3 TM domain. The close proximity of the RRLT sequence to the TM domain, which is required for BNIP3 function, suggests that phosphorylation at this site could alter the activity of the protein. To determine if this putative T188 phosphorylation site could be phosphorylated, we immunoprecipitated His-tagged BNIP3 from HEK 293 cells and probed a Western blot with an α-PKA substrate antibody, which recognizes the sequence RRXS/T only if the S/T residue is phosphorylated. Treatment of cells with 8-Bromo-cAMP increased the amount of BNIP3 detected with this antibody, indicating increased phosphorylation of T188 in response to elevated cAMP (Fig 1A). An increase in T188 phosphorylation of endogenous BNIP3 was also observed upon elevation of cAMP in A549, MDA-MB-231, and AU565 cells, all of which are solid tumor cancer cell lines that express endogenous BNIP3 (Fig 1A) [33, 34]. Furthermore, tandem mass spectrometry of His-tagged BNIP3 purified from HEK 293 cells pretreated with 8-Bromo-cAMP identified multiple C-terminal BNIP3 phosphopeptides, each containing four phosphate groups (Fig 1B and 1C). The limited accuracy of mass spectrometry to identify the specific residues bearing phosphate groups within this cluster of six S/T residues did not allow us to determine the exact combination of S/T residues that were phosphorylated. However, the data does provide reliable evidence that the BNIP3 C-terminus undergoes multiple phosphorylation events in response to cAMP, and supports our evidence of site-specific phosphorylation of T188, detected by Western blot (Fig 1A).

To test the functional relevance of C-terminal BNIP3 phosphorylation, mutations to prevent or mimic site-specific phosphorylation were generated. These included phosphomimetic mutations at one (T188D) or six (6D) C-terminal phosphosites, and the corresponding nonphosphorylated mutants (T188A and 6N, respectively), which cannot be phosphorylated (Fig 1D). The six S/T residues were replaced with asparagine instead of alanine to preserve the hydrophilic and hydrogen bonding nature of the C-terminal region of BNIP3. We also generated a truncated BNIP3 (ΔR), lacking the 10 C-terminal residues (residues 185–194), as well as the dominant negative ΔTM BNIP3, which lacks both the TM domain and the adjacent 10 C-terminal residues (Fig 1D) [35]. Each construct was used to generate a stable doxycycline-inducible (Tet On) HEK 293 cell line (Fig 1E), which does not express endogenous BNIP3 in normoxic conditions [35–37]. The subcellular localization of each BNIP3 mutant was determined, and with the exception of ΔTM BNIP3, all BNIP3 mutants were found associated with mitochondria (Fig 1F). Alkaline extraction of mitochondria determined that ΔR BNIP3 was less tightly associated with mitochondria. However, each BNIP3 phosphomutant and WT BNIP3 remained associated with the mitochondrial membrane after alkaline extraction of mitochondria (Fig 1G).

### C-terminal BNIP3 phosphorylation prevents a loss of mitochondrial content

**Fig 2:**
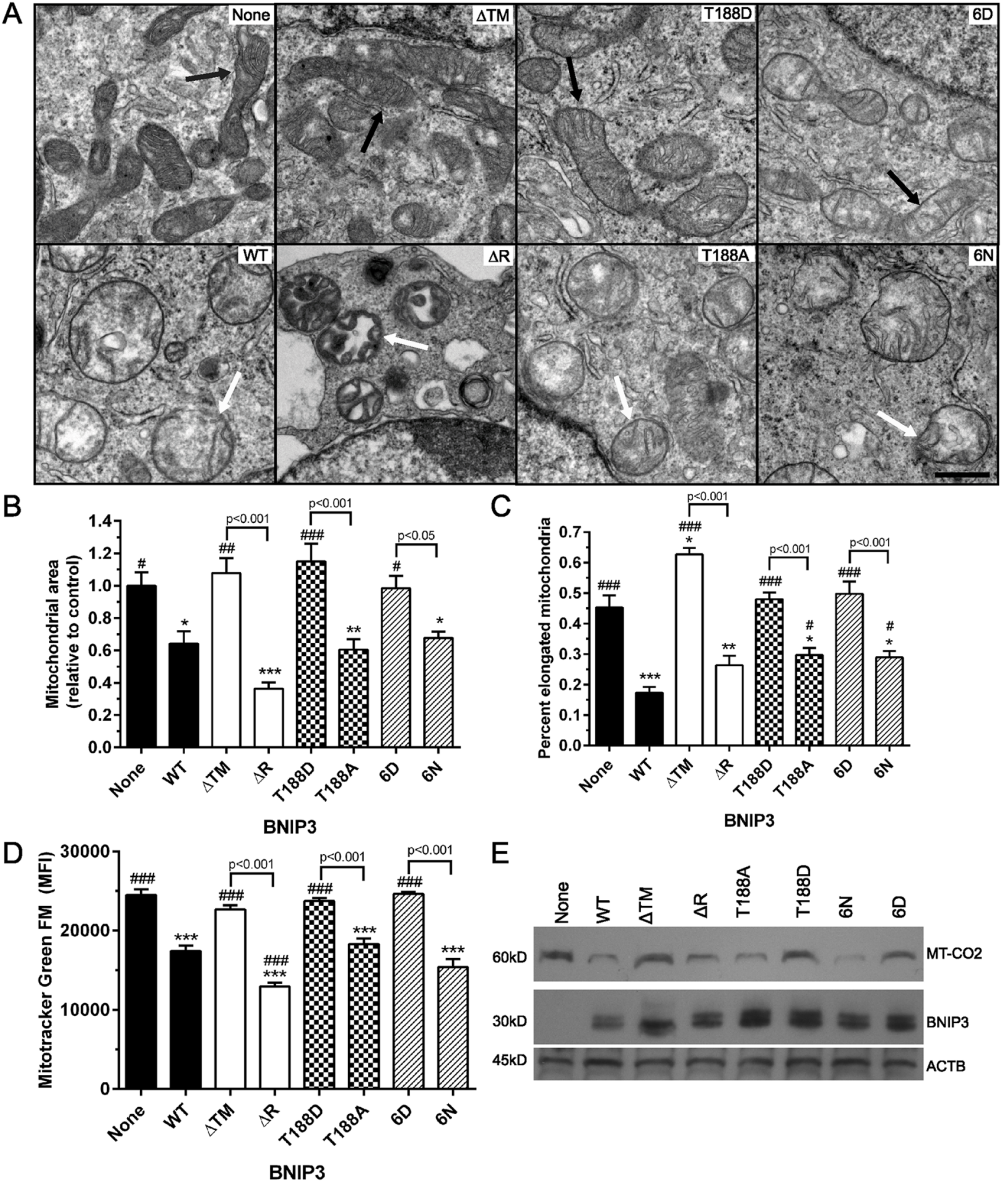
C-terminal BNIP3 phosphorylation prevents damage to the mitochondrial network. (A) Representative examples of mitochondrial morphology, examined via transmission electron microscopy. Black arrows denote healthy, elongated mitochondria and white arrows denote rounded, swollen mitochondria. Scale bar represents 2 μm. (B) Quantification of electron microscopy, showing the average mitochondrial area per field. At least 15 fields were examined per cell type. (C) Percent elongated mitochondria per field, quantified from at least 15 microscope fields per cell type. (D) Mitochondrial mass of HEK 293 cells expressing each BNIP3 mutant, measured by flow cytometric analysis of the mean fluorescence intensity (MFI) of Mitotracker Green FM. (E) Mitochondrial protein levels in HEK 293 cells expressing each BNIP3 mutant, monitored by detection of mitochondrially-encoded cytochrome *c* oxidase subunit II (MT-CO2). For bar graphs, significant differences between control cells (without BNIP3) and cells expressing each BNIP3 mutant are denoted by * p<0.05, ** p<0.01, and *** p<0.001; significant differences between cells expressing WT BNIP3 and either control cells or cells expressing each BNIP3 mutant are denoted by # p<0.05, ## p<0.01, and ### p<0.001; significant differences between complementary pairs of BNIP3 mutants are shown in brackets.

Upon expression of each BNIP3 phosphomutant in HEK 293 cells, mitochondrial morphology and content were examined. Analysis of mitochondrial morphology using transmission electron microscopy revealed a disruption of the mitochondrial network, exemplified by rounding of mitochondria and mitochondrial swelling in cells expressing WT, ΔR, T188A, or 6N BNIP3 (Fig 2A, white arrows). This is consistent with previous studies of WT BNIP3 by electron microscopy [7, 24, 35]. Conversely, the mitochondria of cells expressing T188D or 6D BNIP3 maintained a healthy mitochondrial network, exemplified by the retention of elongated mitochondria (Fig 2A, black arrows). Importantly, comparison of the average mitochondrial area per field and the percent of elongated mitochondria per field revealed that expression of T188D or 6D BNIP3 did not significantly reduce mitochondrial area or increase mitochondrial fragmentation relative to control cells lacking BNIP3 (Fig 2B and 2C). Furthermore, expression of WT or nonphosphorylated ΔR, T188A, or 6N BNIP3 resulted in decreased mitochondrial mass, determined by Mitotracker Green FM fluorescence (Fig 2D). This is consistent with previous observations that WT BNIP3 induces a loss of mitochondrial mass [7]. In contrast, expression of the phosphomimetic T188D or 6D BNIP3 mutants did not significantly reduce mitochondrial mass (Fig 2D). These results were confirmed by Western blot analysis of MT-CO2 (mitochondrially encoded cytochrome *c* oxidase II) levels, where expression of WT or nonphosphorylated BNIP3 reduced MT-CO2 protein levels relative to control cells lacking BNIP3 (Fig 2E).

Finally, mtDNA content of HEK 293 cells expressing each form of BNIP3 was determined by qPCR of ND1 (NADH dehydrogenase subunit 1), where the threshold cycle (Ct) values of ND1 amplification were normalized to B2M (Beta 2 microglobulin) nuclear DNA. Consistent with previous observations of reduced mtDNA content upon expression of WT BNIP3, a significant loss of mtDNA was observed in HEK 293 cells expressing either WT or nonphosphorylated BNIP3 (S1 Fig) [38, 39]. In contrast, the expression of phosphomimetic T188D or 6D BNIP3 did not significantly reduce mtDNA content relative to control cells (S1 Fig). Together, the observed decrease of mtDNA content, mitochondrial area, Mitotracker Green FM fluorescence, and reduced MT-CO2 protein levels in cells expressing WT or nonphosphorylated BNIP3 suggests that BNIP3 with a nonphosphorylated C-terminus continues to function in a manner similar to WT BNIP3. In contrast, the cells expressing phosphomimetic T188D or 6D BNIP3 did not exhibit a significant decrease in mtDNA, mitochondrial area, Mitotracker Green FM fluorescence, or MT-CO2 protein levels, suggesting that phosphorylation of the BNIP3 C-terminus prevents a loss of mitochondrial content (Fig 2 and S1 Fig).

### Mitochondrial damage is inhibited by C-terminal BNIP3 phosphorylation

**Fig 3:**
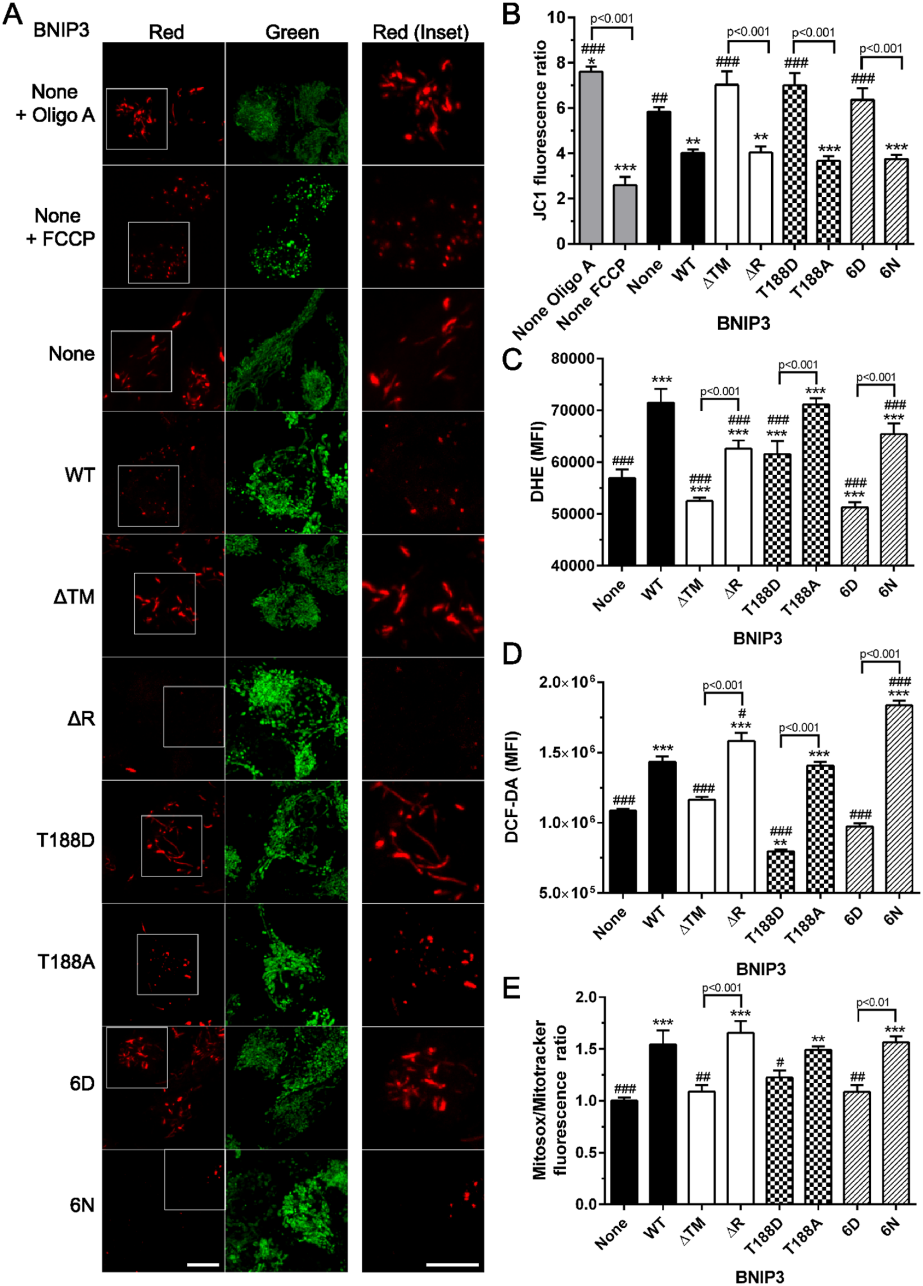
C-terminal BNIP3 phosphorylation inhibits BNIP3-induced loss of mitochondrial function. (A) Representative examples of JC1 dual color fluorescence, examined by confocal microscopy. Red JC1 fluorescence is localized to polarized mitochondria whereas green fluorescence is independent of membrane polarization. Scale bar represents 10 μm. Red JC1 insets, denoted by white outlines, provide examples of the mitochondrial network in cells expressing each BNIP3 phosphomutant. Scale bar for inset is 5μm. Experimental controls included treatment of HEK 293 cells with Oligomycin A (Oligo A) or FCCP to hyperpolarize and depolarize mitochondria, respectively. (B) Mitochondrial membrane potential (ΔΨm), quantified as a ratio of red:green JC1 fluorescence by flow cytometry, where the same positive and negative controls were used to confirm efficacy of the assay (C) Levels of reactive oxygen species (ROS), measured by flow cytometric analysis of DHE fluorescence. (D) ROS levels, quantified by DCF fluorescence. (E) Mitochondrial ROS, measured as a ratio of MitoSox mean fluorescence intensity/Mitotracker mean fluorescence intensity for cells expressing each BNIP3 phosphomutant. All bar graphs represent the results observed in at least 3 independent experiments. Significant differences between control cells (without BNIP3) and cells expressing each BNIP3 mutant are denoted by * p<0.05, ** p<0.01, and *** p<0.001; significant differences between cells expressing WT BNIP3 and cells either lacking BNIP3 (None) or expressing each BNIP3 mutant are denoted by # p<0.05, ## p<0.01, and ### p<0.001; significant differences between pairs of complementary BNIP3 mutants are shown in brackets.

BNIP3 is known to collapse ΔΨm and increase ROS production [3, 22]. To determine the effect of C-terminal BNIP3 phosphorylation on mitochondrial function, cells expressing each form of BNIP3 were probed with JC1, a dual color potentiometric dye which forms red fluorescent aggregates in polarized mitochondria and green fluorescent monomers in nonpolarized cellular compartments. Here, it was observed that expression of WT or nonphosphorylated BNIP3 caused a reduction in JC1 aggregation (red fluorescence) comparable to the reduced JC1 red fluorescence observed upon treatment of control cells with FCCP, a mitochondrial uncoupler. Conversely, expression of the phosphomimetic T188D or 6D BNIP3 mutants did not reduce red JC1 fluorescence, indicating a maintenance of polarized mitochondria in these cells (Fig 3A). The maintenance of polarized mitochondria in cells expressing T188D or 6D BNIP3 was confirmed by quantification of the number of JC1 red puncta per cell, in which the number of red JC1 puncta was not significantly different from control cells lacking BNIP3 (S2A Fig). This pattern was also observed upon analysis of JC1 fluorescence by flow cytometry, where the ratio of red:green JC1 fluorescence serves as an indicator of the ratio of polarized:depolarized mitochondria (Fig 3B). Importantly, expression of WT or nonphosphorylated BNIP3 mutants reduced mitochondrial membrane potential to a level similar to HEK 293 cells treated with FCCP. In contrast, the mitochondrial membrane potential of cells expressing T188D or 6D BNIP3 was not significantly different from cells expressing no BNIP3, and remained similar to the mitochondrial membrane potential of control cells treated with Oligomycin A (Oligo A), which causes mitochondrial hyperpolarization [40]. Finally, mitochondrial membrane potential was monitored by DiOC6 fluorescence, which accumulates in polarized mitochondria [40]. Consistent with the patterns observed using JC1 dual color fluorescence, cells expressing WT or nonphosphorylated BNIP3 exhibited significantly lower DiOC6 fluorescence compared to control cells, indicating a loss of mitochondrial membrane potential. However, the phosphomimetic T188D and 6D BNIP3 mutants did not significantly reduce DiOC6 fluorescence relative to control cells, suggesting that these cells maintain high mitochondrial membrane potential (S2B Fig).

In addition to the loss of ΔΨm, fragmentation of the mitochondrial network was visible in cells expressing WT, ΔR, T188A, or 6N BNIP3 (Fig 3A, red inset). This result is consistent with our observations that WT and nonphosphorylated BNIP3 mutants decrease the percent of elongated mitochondria (Fig 2C), as well as previous reports that WT BNIP3 induces mitochondrial fragmentation via increased fission events [14]. Since collapse of ΔΨm can lead to the generation of excess reactive oxygen species, we determined levels of ROS using a series of fluorescent probes. Consistent with previous evidence of increased ROS upon expression of WT BNIP3, HEK 293 cells expressing WT or nonphosphorylated BNIP3 significantly increased whole cell ROS, as measured by DHE and DCF fluorescence (Fig 3C and 3D) [3]. Additionally, the nonphosphorylated BNIP3 mutants significantly increased mitochondrial ROS, quantified by Mitosox fluorescence (Fig 3E). Representative flow cytometry histograms of each ROS probe are provided in S3 Fig. As predicted by their lack of effect on ΔΨm, the phosphomimetic T188D and 6D BNIP3 mutants did not significantly increase mitochondrial ROS (Fig 3E).

### C-terminal BNIP3 phosphorylation does not prevent autophagy

**Fig 4:**
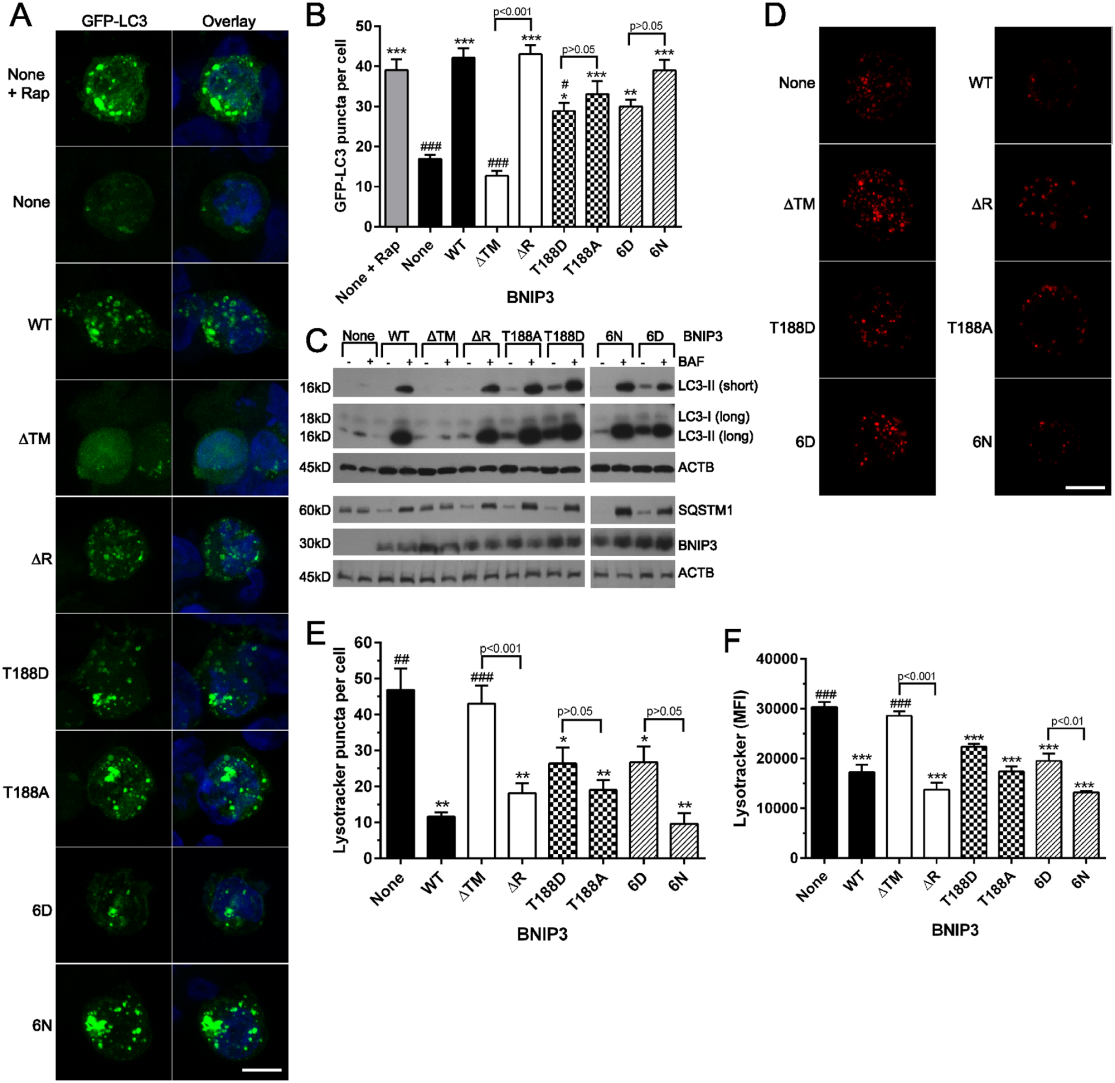
C-terminal BNIP3 phosphorylation does not inhibit BNIP3-induced autophagy. (A) Representative images of GFP-LC3 puncta in HEK 293 cells expressing each BNIP3 mutant, examined via confocal microscopy. At least 50 cells were examined per cell type, in 3 independent experiments. Rapamycin (Rap) was used as a positive control. Scale bar represents 10 μm. (B) Quantification of the number of GFP-LC3 puncta per cell. (C) Western blot analysis of autophagic flux, where cells expressing each BNIP3 phosphomutant were treated without or with 50 nM Bafilomycin A1 (BAF). Blots are representative of 4 independent experiments. Two exposures of the LC3 Western blot are provided to show levels of LC3-II (short exposure) and LC3-I (long exposure). (D) Representative images of HEK 293 cells probed with Lysotracker Red, examined by confocal microscopy. (E) Quantification of the number of lysotracker puncta per cell, where a minimum of 30 cells were examined in 3 independent experiments. Scale bar represents 10 μm. (F) Quantification of mean lysotracker fluorescence intensity, measured by flow cytometry in 3 independent experiments. For bar graphs, significant differences between control cells (without BNIP3) and cells expressing each BNIP3 mutant are denoted by * p<0.05, ** p<0.01, and *** p<0.001; significant differences between cells expressing WT BNIP3 and either control cells or cells expressing each BNIP3 mutant are denoted by # p<0.05, ## p<0.01, and ### p<0.001; significant differences between complementary pairs of BNIP3 mutants are shown in brackets.

BNIP3 has a well-established role in promoting autophagy [28]. Therefore, we determined the level of autophagy activation in HEK 293 cells expressing each C-terminal BNIP3 phosphomutant. With the exception of ΔTM BNIP3, which is known to be defective in promoting autophagy [6], cells expressing each form of BNIP3 exhibited an increased number of GFP-LC3 puncta per cell (Fig 4A). Quantification of the number of GFP-LC3 puncta per cell confirmed this phenotype, where WT and ΔR BNIP3 stimulated formation of LC3 puncta to the same extent as treatment with rapamycin, a well-known stimulator of autophagy that acts on the mTOR pathway (Fig 4B) [41]. Further, the nonphosphorylated T188A and 6N BNIP3 mutants stimulated LC3 puncta formation to levels similar to that of WT or ΔR BNIP3. In addition, both T188D and 6D BNIP3 significantly increased LC3 puncta formation relative to control cells not expressing BNIP3. Importantly, although fewer GFP-LC3 puncta were observed in cells expressing T188D or 6D BNIP3 relative to the corresponding nonphosphorylated BNIP3 mutants, these differences were not significant (Fig 4B), suggesting that autophagy activation is not blocked by C-terminal BNIP3 phosphorylation.

Next, we assessed autophagic flux using Western blot detection of LC3 and SQSTM1 in cells treated with or without the lysosomal acidification inhibitor Bafilomycin A1 (BAF), which blocks autophagic flux and prevents the degradation of LC3-II and SQSTM1 [42]. Consistent with the results of GFP-LC3 puncta formation, cells expressing each BNIP3 phosphomutant exhibited an increase in LC3-II and SQSTM1 upon treatment with BAF (Fig 4C). Additionally, relative to control cells lacking BNIP3, the ratio of LC3-II in BAF treated cells/LC3-II in untreated cells increased upon expression of WT or phosphomutant BNIP3 (S4A Fig). Similar results were observed with respect to SQSTM1 protein levels (S4B Fig). Together, these results are consistent with previous observations of autophagy activation upon expression of WT BNIP3, and suggest that expression of each BNIP3 phosphomutant increases autophagic flux, which is the turnover of autophagosomes due to autophagy [8, 11]. Importantly, previous evidence has demonstrated that expression of BNIP3 does not alter LC3 nor SQSTM1 transcription, suggesting that the differences in protein levels observed here are due to protein degradation and not due to altered transcription levels [8]. To determine whether the time course of autophagy activation differed between cells expressing the panel of BNIP3 phosphomutants, GFP-LC3 puncta formation was observed 24, 48, and 72 hr after induction of BNIP3 expression. Consistent with previous observations, each BNIP3 phosphomutant significantly increased the number of GFP-LC3 puncta at each time point relative to control cells (S4 Fig). Together, these observations suggest that BNIP3-induced autophagic flux is not altered by phosphorylation at the C-terminus.

The limiting factor for autophagosome processing in cells expressing BNIP3 is the availability of lysosomes, which are consumed following fusion with autophagosomes [8]. To confirm the autophagic phenotype of cells expressing BNIP3 phosphomutants, the availability of lysosomes was quantified. Confocal microscopy of HEK 293 cells probed with Lysotracker Red revealed a significant decrease of the lysosomal population in cells expressing WT, ΔR, T188A, or 6N BNIP3 (Fig 4D). Expression of phosphomimetic T188D or 6D BNIP3 also significantly decreased the lysosome population in HEK 293 cells, but to a slightly lesser extent than WT BNIP3 or the nonphosphorylated BNIP3 mutants. However, the differences between these forms of BNIP3 were not significant (WT BNIP3 vs T188D or 6D BNIP3) (Fig 4E). These results were confirmed with Lysotracker fluorescence analyzed by flow cytometry, where each BNIP3 phosphomutant significantly decreased lysotracker fluorescence (Fig 4F). Importantly, the differences in lysosome populations among cells expressing WT, T188D or 6D BNIP3 were not significant, providing evidence that C-terminal phosphorylation does not block BNIP3-induced autophagy (Fig 4F).

Despite the lack of mitochondrial damage in cells expressing T188D or 6D BNIP3, these phosphomutants continue to activate autophagy. This could occur due to the decoration of mitochondria with the BNIP3 phosphomutants, which provide a link to LC3-II on nascent autophagosomes and increase mitophagy [11, 12]. Additionally, BNIP3 can activate macroautophagy through its interaction with BCL2, which causes the release of BECN1from BCL2 and promotes BECN1-mediated autophagosome synthesis [28, 43, 44]. Importantly, previous evidence indicates that BNIP3-induced autophagy depends on BECN1 in both normoxic and hypoxic conditions [6, 12, 28, 38, 45]. To address whether C-terminal BNIP3 phosphorylation alters this mechanism of autophagy activation, the ability of each BNIP3 phosphomutant to interact with BCL2 was determined by co-immunoprecipitation. Both phosphomimetic and nonphosphorylated BNIP3 mutants maintained their interaction with BCL2, suggesting that this pathway of autophagy activation is intact (S5 Fig).

### C-terminal BNIP3 phosphorylation regulates the activation of cell death

**Fig 5:**
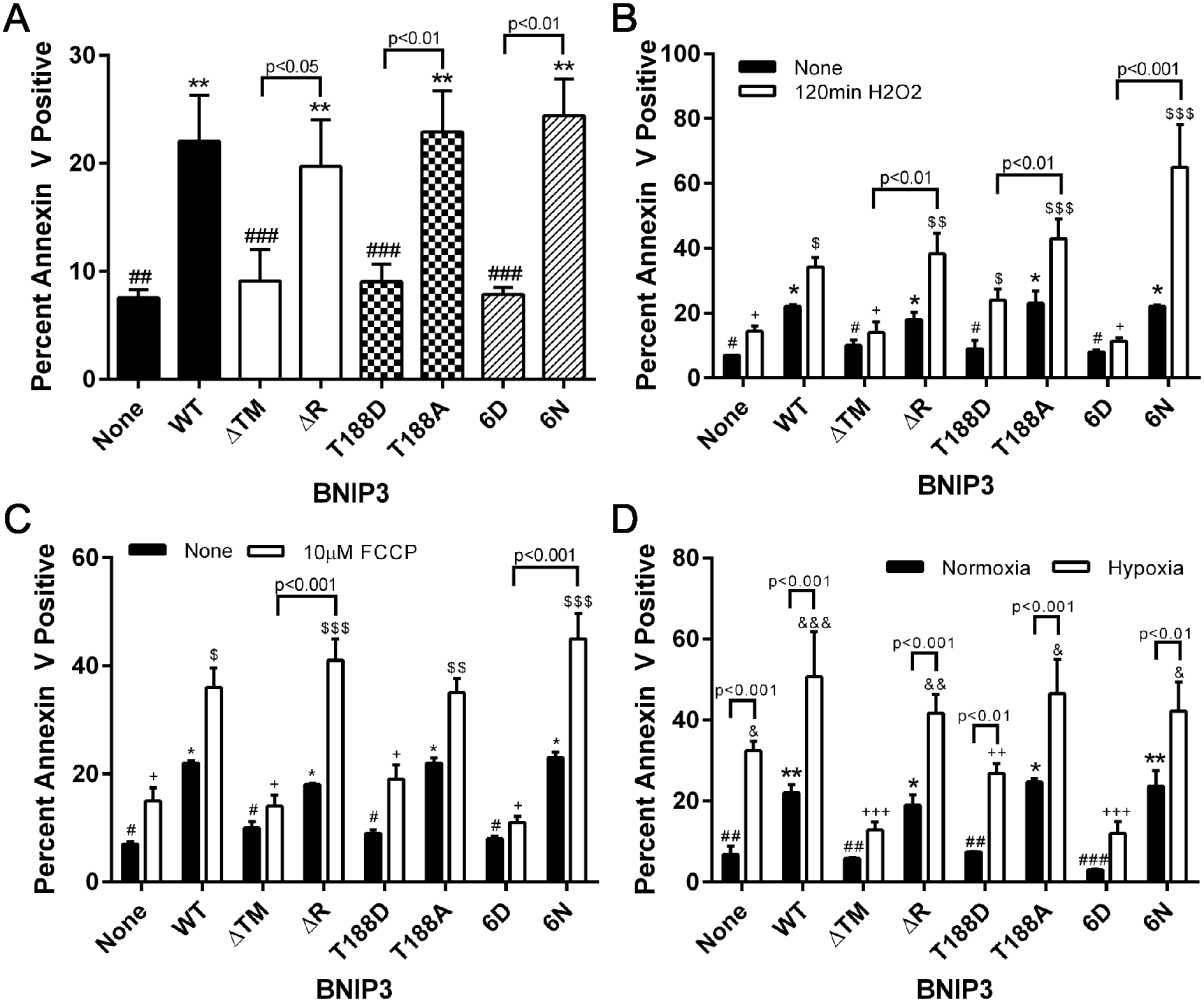
C-terminal BNIP3 phosphorylation inhibits BNIP3-induced cell death. (A) Quantification of the percent of Annexin V positive cells 24 hr after induction of BNIP3 expression, analyzed by flow cytometry. Data is expressed as the average of 4 independent experiments. Significant differences between control cells (no BNIP3) and cells expressing each form of BNIP3 are denoted by * p<0.05, ** p<0.01, and *** p<0.001; significant differences between complementary pairs of BNIP3 mutants are shown in brackets. (B), (C), and (D): Percent Annexin V positive cells expressing WT or phosphomutant BNIP3 with or without the following treatments: (B) 150μM H_2_O_2_ for 120 min, (C) 10μM FCCP for 120 min, and (D) 48 hr hypoxia. In (B) and (C), significant differences between control cells (without BNIP3) and cells expressing each BNIP3 mutant in normal conditions are denoted by * p<0.05, ** p<0.01, and *** p<0.001; significant differences between control cells and cells expressing each BNIP3 mutant, all undergoing additional cellular stress (H_2_O_2_, or FCCP), are denoted by $ p<0.05, $ $ p<0.01, and $ $ $ p<0.001. Significant differences between cells expressing WT BNIP3 and cells either lacking BNIP3 (None) or expressing each BNIP3 mutant in normal conditions are denoted by # p<0.05, ## p<0.01, and ### p<0.001; significant differences between cells expressing WT BNIP3 and cells either lacking BNIP3 (None) or expressing each BNIP3 mutant in the presence of H_2_O_2_ or FCCP are denoted by + p<0.05, ++ p<0.01, and +++ p<0.001; significant differences between complementary pairs of BNIP3 mutants treated with H_2_O_2_ or FCCP are shown in brackets. In (D), significant differences between control cells (without BNIP3) and cells expressing each BNIP3 mutant in normoxia are denoted by * p<0.05, ** p<0.01, and *** p<0.001; significant differences between cells expressing ΔTM BNIP3 and cells expressing each other form of BNIP3, all cultured in hypoxia, are denoted by & p<0.05, && p<0.01, and &&& p<0.001. Significant differences between cells expressing WT BNIP3 and cells either lacking BNIP3 (None) or expressing each BNIP3 mutant in normal conditions are denoted by # p<0.05, ## p<0.01, and ### p<0.001; significant differences between cells expressing WT BNIP3 and cells either lacking BNIP3 (None) or expressing each BNIP3 mutant in hypoxic conditions are denoted by + p<0.05, ++ p<0.01, and +++ p<0.001; significant differences between treatment conditions in cells expressing the same BNIP3 mutant are shown in brackets.

Given the differential ability of phosphomimetic and nonphosphorylated C-terminal BNIP3 mutants to damage mitochondria, levels of cell death in HEK 293 cells expressing each BNIP3 phosphomutant were measured by Annexin V fluorescence. Consistent with previous reports of increased cell death upon expression of WT or ΔR BNIP3, the percent of Annexin V positive cells was highest in HEK 293 cells expressing WT or nonphosphorylated BNIP3 (Fig 5A) [9, 32]. Conversely, expression of ΔTM, T188D, or 6D BNIP3 did not significantly increase cell death (Fig 5A). Thus, under normal conditions, C-terminal BNIP3 phosphorylation prevents cell death while allowing autophagy to proceed. Levels of HEK 293 cell death were also monitored upon exposure to stress conditions. The addition of H_2_O_2_ or FCCP to cells was chosen to increase cellular oxidative stress and mitochondrial stress, respectively. Upon addition of H_2_O_2_, cells expressing WT or nonphosphorylated BNIP3 exhibited significant increases in Annexin V fluorescence, whereas cells expressing 6D BNIP3 exhibited full protection from H_2_O_2_ toxicity (Fig 5B). In contrast, cells expressing T188D BNIP3 exhibited an intermediate level of cell death during H_2_O_2_-induced cellular stress, with cell death being significantly increased compared to H_2_O_2_-stressed control cells (not expressing BNIP3), but still significantly lower compared to the level of cell death observed in the H_2_O_2_-treated cells expressing the complementary T188A BNIP3 mutant (Fig 5B). Similar results were observed upon mitochondrial stress using FCCP (Fig 5C).

To determine whether the BNIP3 phosphomutants alter the function of endogenous (WT) BNIP3, HEK 293 cells expressing each BNIP3 phosphomutant were exposed to hypoxia for 48 hr to induce endogenous BNIP3 [26, 35], and cell death was quantified by Annexin V fluorescence. Hypoxic cells expressing WT or nonphosphorylated BNIP3 exhibited the highest levels of cell death (Fig 5D). Notably, a significant increase in cell death was observed in hypoxic control cells (no exogenous BNIP3), reflecting expression of endogenous BNIP3 during hypoxia [36, 38]. As expected, co-expression of endogenous WT BNIP3 with exogenous ΔTM BNIP3 prevented hypoxia-induced cell death due to the known action of ΔTM BNIP3 as a dominant negative form of BNIP3 [33, 36]. Interestingly, cells expressing 6D BNIP3 were protected from the effect of endogenous BNIP3, suggesting that this phosphomimetic BNIP3 mutant blocks the cytotoxic effects of WT BNIP3 in a manner similar to dominant negative ΔTM BNIP3 (Fig 5D). In contrast, cells expressing T188D BNIP3 exhibited significantly increased levels of death during hypoxia relative to cells expressing T188D BNIP3 in normoxic conditions. However, the level of death observed in the hypoxic T188D BNIP3 cells was not significantly different from cells expressing ΔTM BNIP3 in hypoxia, and remained significantly lower than the level of death observed in cells expressing WT BNIP3 during hypoxia (Fig 5D). Importantly, cells expressing T188D BNIP3 during hypoxia exhibited lower levels of cell death relative to cells expressing the complementary T188A BNIP3 phosphomutant during hypoxia, suggesting that T188D BNIP3 can partly counteract the toxicity of endogenous WT BNIP3. Together, this data suggests that while 6D BNIP3 appears to offer full protection from the toxicity of endogenous BNIP3, T188D BNIP3 offers partial protection from stress-induced cell death.

### The BNIP3-OPA1 interaction is reduced by C-terminal BNIP3 phosphorylation

**Fig 6:**
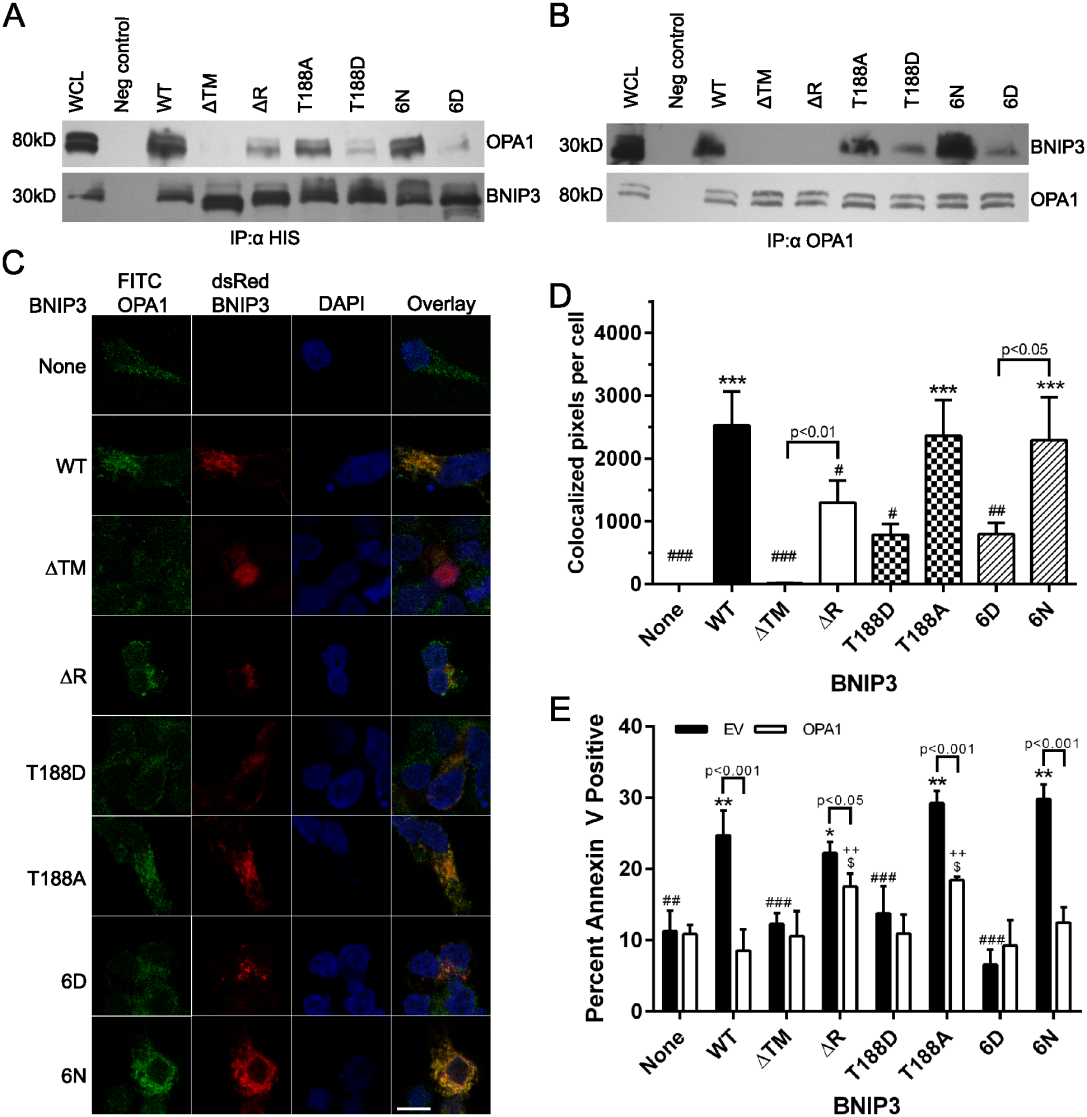
C-terminal BNIP3 phosphorylation decreases the BNIP3-OPA1 interaction at the mitochondrial membrane. (A) Detection of OPA1 by Western blot following immunoprecipitation of BNIP3 with an α-His tag antibody. (B) Reverse co-immunoprecipitation in which OPA1 was immunoprecipitated (α-OPA1 antibody), and co-immunoprecipitated BNIP3 was detected by Western blot. Blots are representative of 3 experiments. WCL = whole cell lysate. (C) Colocalization of BNIP3 and OPA1 at the mitochondria, examined by confocal microscopy following transient OPA1 overexpression. A minimum of 30 cells expressing each BNIP3 mutant were examined in 3 independent experiments. Scale bar represents 10 μm. (D) Quantification of BNIP3-OPA1 colocalization, expressed as the number of colocalized BNIP3-OPA1 pixels per cell. Significance of each cell type vs control cells (no BNIP3) is denoted by *** p<0.001; significance of each cell type vs cells expressing WT BNIP3 is denoted by # p<0.05, ## p<0.01, and ### p<0.001. (E) Percent Annexin V positive cells expressing BNIP3 with either normal levels of OPA1 (transfected with empty vector, EV) or high levels of OPA1 (transfected with OPA1) for 24 hr. Significance of each cell type vs control cells (no BNIP3) with normal levels of OPA1 is denoted by * p<0.05, ** p<0.01, and *** p<0.001; significant differences between each cell type overexpressing OPA1 vs control cells (no BNIP3) overexpressing OPA1 is denoted by $ p<0.05. Significant differences between cells expressing WT BNIP3 with cells not expressing BNIP3 (None) or expressing each BNIP3 mutant, all with normal levels of OPA1 is denoted by # p<0.05, ## p<0.01, and ### p<0.001; significant differences between each cell type overexpressing OPA1 vs cells expressing WT BNIP3 with exogenous OPA1 are denoted by ++ p<0.01; significant differences between conditions (normal levels of OPA1 vs OPA1 overexpression) for each cell type are denoted in brackets.

BNIP3 and OPA1 have been shown to interact in the intermembrane space of mitochondria, in a manner dependent on the extreme C-terminus of BNIP3 [16]. To determine whether C-terminal BNIP3 phosphorylation regulates the interaction of BNIP3 with OPA1, co-immunoprecipitation assays were performed. Due to the reduced level of OPA1 present in HEK 293 cells expressing WT or nonphosphorylated BNIP3, the co-immunoprecipitation assays were performed using HEK 293 cells expressing each BNIP3 mutant with simultaneous OPA1 overexpression, thus minimizing differences in OPA1 availability between cell types (S6A Fig). Following immunoprecipitation of BNIP3 using an α-His tag antibody, OPA1 was detected by Western blot. The highest amounts of co-immunoprecipitated OPA1 were detected in cells expressing WT, T188A, or 6N BNIP3 (Fig 6A). Consistent with previous reports, a markedly decreased amount of OPA1 co-immunoprecipitated with ΔR BNIP3, which lacks the extreme C-terminus (Fig 1D) [16]. Importantly, the lowest amount of OPA1 was co-immunoprecipitated from cells expressing T188D or 6D BNIP3, suggesting that C-terminal BNIP3 phosphorylation decreases the BNIP3-OPA1 interaction (Fig 6A). The decreased interaction between ΔR, T188D, or 6D BNIP3 with OPA1 was confirmed using the reverse co-immunoprecipitation assay, in which an α-OPA1 antibody co-immunoprecipitated low levels of ΔR, T188D, and 6D BNIP3 and higher levels of WT, T188A, and 6N BNIP3 (Fig 6B). In both co-immunoprecipitation experiments, ΔTM BNIP3 did not interact with OPA1, as expected (Fig 6A and 6B). Additionally, the colocalization of BNIP3 and OPA1 was observed via confocal microscopy, where WT, T188A and 6N BNIP3 exhibited strong colocalization with OPA1, and ΔR, T188D, and 6D BNIP3 had decreased colocalization with OPA1 (Fig 6C). Quantification of the number of colocalized BNIP3-OPA1 pixels per cell confirmed that ΔR, T188D, and 6D BNIP3 exhibited significantly decreased colocalization with OPA1 relative to WT BNIP3 (Fig 6D). To ensure the accuracy of these observations, the colocalization of WT or mutant BNIP3 with OPA1 at mitochondria was also monitored using simultaneous detection of MT-CO2, BNIP3, and OPA1 by confocal microscopy (S7 Fig).

The BNIP3-OPA1 interaction has been shown to promote fragmentation of the mitochondrial network and induce cell death [15, 16]. Importantly, this effect could be abrogated by increased levels of OPA1 [16]. To address whether the expression of excess OPA1 could protect cells from death caused by WT, T188A, or 6N BNIP3, cells expressing each BNIP3 mutant were transiently transfected with OPA1, and Annexin V fluorescence measured 24 hr post-transfection. Overexpression of OPA1 along with WT BNIP3 substantially decreased cell death (Fig 6E), consistent with the literature [16]. Furthermore, cell death significantly decreased upon OPA1 overexpression in cells expressing T188A or 6N BNIP3, but levels of death did not decrease in cells expressing phosphomimetic T188D or 6D BNIP3 (Fig 6E). Together, these experiments indicate that only BNIP3 with a nonphosphorylated C-terminal domain can strongly interact with OPA1, leading to mitochondrial fragmentation and cell death.

### Modulation of C-terminal BNIP3 phosphorylation occurs in physiological conditions

**Fig 7:**
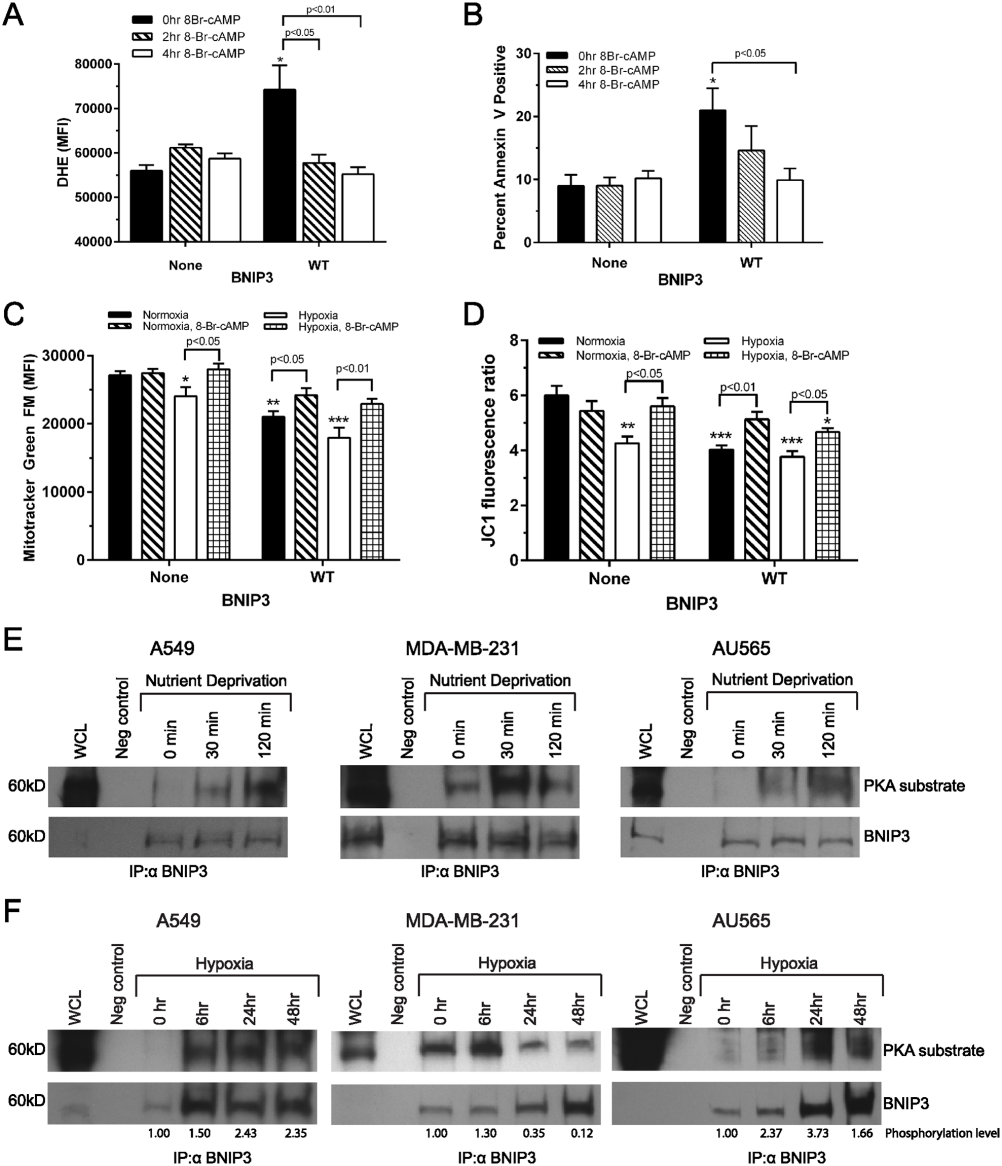
C-terminal BNIP3 phosphorylation can be modulated by cellular stresses. (A) Levels of ROS, measured by the mean fluorescence intensity of DHE. HEK 293 control cells and cells expressing WT BNIP3 for 48 hr were treated with 8-Br-cAMP for 0, 2, or 4hr immediately prior to analyzing DHE fluorescence by flow cytometry. (B) Percent Annexin V positive cells undergoing the same 8-Br-cAMP treatment as described in (A). (C) Mitochondrial mass of HEK 293 cells cultured in normoxia or hypoxia for 48 hr and with or without 8-Br-cAMP treatment for the last 6 hr of normoxia/hypoxia. (D) Mitochondrial membrane potential of cells treated as described in (C). For each assay, a minimum of 30,000 events were collected per sample by flow cytometry. Data represents results from at least 3 separate experiments. Significant differences between untreated control cells (without BNIP3) and cells expressing WT BNIP3 are denoted by * p<0.05, ** p<0.01, and *** p<0.001; significant differences between treatment conditions are shown in brackets. (E) Phosphorylation of endogenous BNIP3 at T188 following nutrient deprivation for 0, 30, or 120 min in three cell types: A549, MDA-MB-231, and AU565 cells, detected by probing immunoprecipitated BNIP3 with an α-PKA substrate antibody. (F) Phosphorylation level of endogenous BNIP3 at T188 following hypoxia for 0, 6, 24, or 48 hr in three cell types: A549, MDA-MB-231, and AU565 carcinoma cells, detected by probing immunoprecipitated BNIP3 with an α-PKA substrate antibody. To account for the increased expression of BNIP3 during extended hypoxia, the relative level of T188 BNIP3 phosphorylation is provided below each lane. The phosphorylation level, which represents the ratio of BNIP3 detected by α-PKA substrate antibody/total BNIP3, is expressed relative to the 0 hr time point of each cell type.

Due to the observed increase in C-terminal BNIP3 phosphorylation upon cAMP elevation, we examined whether the function of WT BNIP3 was altered following elevation of cAMP. To address this question, HEK 293 cells expressing WT BNIP3 for 48 hr were treated with 8-Br-cAMP for 2 or 4 hr, followed by fluorescence detection of ROS and cell death. Elevation of cAMP significantly decreased levels of both ROS and Annexin V positivity in cells expressing WT BNIP3 relative to untreated WT BNIP3 cells (Fig 7A and 7B). This is consistent with our observations that cAMP increases C-terminal BNIP3 phosphorylation and that phosphomimetic mutation of the C-terminus prevents BNIP3 toxicity (Figs 1–3). Next, HEK 293 control and WT BNIP3 cells were subjected to normoxia or hypoxia for 48 hr with or without cAMP elevation for the last 6 hr of normoxia/hypoxia. Cells expressing WT BNIP3 and exposed to hypoxia exhibited a significant loss of mitochondrial mass and ΔΨm, but upon addition of 8-Br-cAMP, these cells exhibited partial rescue of both mitochondrial mass and ΔΨm (Fig 7C and 7D). These results are consistent with the observed inhibition of BNIP3 cytotoxicity upon phosphomimetic mutation of the BNIP3 C-terminus (Figs 2, 3 and 5).

Two of the conditions in which BNIP3 expression and toxicity increase are nutrient deprivation and hypoxia, both of which frequently occur in solid tumor cells [46]. To further define the physiological conditions in which C-terminal BNIP3 phosphorylation occurs, BNIP3 phosphorylation at residue T188 was monitored during each of these stresses. First, A549, MDA-MB-231, and AU565 carcinoma cells, all of which express endogenous BNIP3, were subjected to nutrient deprivation for 0, 30, or 120 min. Following immunoprecipitation of endogenous BNIP3, Western blots were probed with an α-PKA substrate antibody to detect levels of BNIP3 T188 phosphorylation. In each cell type, the amount of BNIP3 T188 phosphorylation increased (Fig 7E), suggesting that C-terminal BNIP3 phosphorylation can be modulated by nutrient deprivation. Second, T188 phosphorylation of endogenous BNIP3 was monitored in the same three carcinoma cell lines following exposure to hypoxia for 0, 6, 24, or 48 hr. To account for the increased BNIP3 expression during long-term hypoxia, the ratio of T188 phosphorylated BNIP3/total BNIP3 is provided below each Western blot. In each case, exposure to hypoxia increased T188 BNIP3 phosphorylation. However, while the breast carcinoma MDA-MB-231 and AU565 cells exhibited a transient increase in T188 phosphorylation, T188 BNIP3 phosphorylation increased and remained high during extended hypoxia in lung carcinoma A549 cells (Fig 7F). Together, this data suggests that while C-terminal BNIP3 phosphorylation can be modulated in physiologically-relevant conditions, the use of this mechanism to regulate BNIP3 function in short-term and long-term stress conditions may differ by cell type and microenvironment.

## Discussion

Here we present evidence that phosphorylation of the BNIP3 C-terminus prevents mitochondrial damage and cell death while still allowing a significant level of autophagy to proceed. The identification of several C-terminal BNIP3 phosphorylation sites adds to our understanding of how BNIP3 is post-translationally regulated in the cell. The presence of a canonical PKA recognition sequence, along with evidence that elevated cAMP increases C-terminal BNIP3 phosphorylation, suggests that the BNIP3 C-terminus might be phosphorylated by cAMP-dependent kinases, which are known to regulate local signaling dynamics at mitochondria (Fig 1) [47–49]. However, it is likely that other kinases are also involved to achieve the multiply phosphorylated species observed at the BNIP3 C-terminus.

Upon expression of C-terminal phosphomimetic or nonphosphorylated BNIP3 mutants in HEK 293 cells, several distinct phenotypes were observed. Expression of WT, ΔR, T188A, or 6N BNIP3 caused a significant fragmentation of the mitochondrial network and loss of mitochondrial mass, as well as a significant loss of mitochondrial function (Figs 2 and 3). These phenotypes suggest that nonphosphorylated BNIP3 retains its ability to damage mitochondria. In contrast, expression of phosphomimetic T188D or 6D BNIP3 did not significantly reduce mitochondrial function, demonstrating that despite their localization to the mitochondrial membrane, these BNIP3 phosphomutants are unable to damage mitochondria (Figs 2 and 3). This suggests that phosphorylation of C-terminal BNIP3 S/T residues blocks the cytotoxic effects of BNIP3 by inhibiting mitochondrial damage.

With respect to the activation of autophagy, it was determined that each BNIP3 phosphomutant significantly increased levels of autophagy (Fig 4). Despite the lack of mitochondrial damage in cells expressing phosphomimetic BNIP3, autophagy was activated in a BNIP3-dependent manner, suggesting that the protein can activate autophagy via a mechanism independent of mitochondrial damage. BECN1 (Beclin 1) is a key regulator of autophagosome synthesis, thus regulating the activation of autophagy [43, 44]. One well-established inhibitor of BECN1-mediated autophagy activation is BCL2, which interacts with BECN1 and causes it to adopt an inactive state [50]. It has also been established that in the presence of BNIP3, BCL2 selectively interacts with BNIP3 and releases BECN1 from its inactive state [9, 38, 51]. Upon release from BCL2, BECN1 becomes available to interact with PtdIns3K (phosphatidylinositol 3-kinase) to induce autophagosome formation [43, 52]. Here, we provide evidence that C-terminal BNIP3 phosphorylation does not prevent BNIP3 from interacting with BCL2, consistent with evidence demonstrating that this protein-protein interaction is dependent on the BNIP3 TM domain but not the extreme BNIP3 C-terminus (S5 Fig) [9]. This suggests that C-terminal BNIP3 phosphorylation does not alter the ability of BNIP3 to promote BECN1-mediated autophagy, and is consistent with evidence that BNIP3 induces BECN1-dependent autophagy before mitochondrial damage occurs [10, 12, 38].

In addition to activating autophagy via its interactions with BCL2, BNIP3 can promote mitophagy via signals generated by mitochondrial damage and the BNIP3-LC3-II interaction. Following BNIP3-induced mitochondrial damage, both DRP1 and PARK2 are recruited to mitochondria with low ΔΨm, where they increase mitochondrial fission and promote mitophagy [14, 53]. This suggests that WT, ΔR, T188A, and 6N BNIP3 activate higher levels of autophagy than the complementary phosphomimetic T188D and 6D BNIP3 mutants due to their ability to damage mitochondria. However, since each BNIP3 phosphomutant localizes to mitochondria, C-terminal BNIP3 phosphorylation is unlikely to alter the BNIP3-LC3-II interaction, which is dependent on the LIR near the BNIP3 N-terminus [11, 12]. Thus, while nonphosphorylated BNIP3 likely activates mitophagy through mitochondrial damage-associated signals, the protein retains its ability to activate macroautophagy via modulation of the BCL2-BECN1 interaction independent of C-terminal BNIP3 phosphorylation.

Consistent with the mitochondrial damage observed in cells expressing WT or nonphosphorylated BNIP3 mutants, levels of cell death also significantly increased. In addition, when cells were subjected to stress conditions, including H_2_O_2_ or FCCP treatment or hypoxia, the cells expressing WT or nonphosphorylated BNIP3 exhibited increased susceptibility to stress-induced cell death compared to control cells exposed to the same stress conditions. However, cells expressing 6D BNIP3 exhibited protection from cell death even under stress conditions, suggesting that a fully phosphorylated BNIP3 C-terminus can block the cytotoxic effects of BNIP3 during oxidative stress or hypoxia. Additionally, cells expressing T188D BNIP3 exhibited partial protection from both oxidative stress and hypoxia (Fig 5B–5D). Together, these observations suggest that while T188D BNIP3 offers partial protection from damage caused to the cell during stress conditions, 6D BNIP3 offers complete protection from BNIP3-induced cell death in both normal and stress conditions. Finally, the observation that cell death was higher in control cells exposed to hypoxia, in which endogenous BNIP3 is expressed, compared to T188D or 6D BNIP3 cells exposed to hypoxia, in which both phosphomimetic and endogenous BNIP3 are expressed, suggests that both the T188D and 6D BNIP3 have the ability to block the cytotoxicity of endogenous BNIP3, similar to the dominant negative effect of ΔTM BNIP3 (Fig 5D).

The BNIP3-OPA1 interaction in the intermembrane space has been suggested to regulate BNIP3-induced cell death [16]. Here we showed that C-terminal BNIP3 phosphorylation alters the extent of the BNIP3-OPA1 interaction. Co-immunoprecipitation assays confirmed that phosphomimetic T188D and 6D BNIP3 exhibit weaker interactions with OPA1 than the nonphosphorylated T188A and 6N BNIP3 (Fig 6A and 6B). The interaction between T188D or 6D BNIP3 with OPA1 was also decreased compared to ΔR BNIP3, which lacks part of the BNIP3 C-terminus important for the protein interaction [16]. The strong colocalization of 6N BNIP3 with OPA1 and the weaker colocalization of 6D BNIP3 with OPA1 also provides evidence that this interaction is reduced specifically by negative charge at the C-terminus, present when the BNIP3 C-terminus is phosphorylated (Fig 6C and 6D).

The loss of the BNIP3-OPA1 interaction upon BNIP3 phosphorylation can also explain the impaired ability of C-terminally phosphorylated BNIP3 to induce cell death. Landes et al. previously showed that the BNIP3-OPA1 interaction promotes mitochondrial fragmentation and cell death [16]. Our data demonstrates that C-terminal BNIP3 phosphorylation reduces the interaction with OPA1, decreases mitochondrial damage, and prevents cell death. Combined with evidence that the BNIP3-OPA1 interaction promotes mitochondrial fragmentation and cell death, these observations suggest that although the phosphomimetic BNIP3 mutants localize to the mitochondria, their decreased interaction with OPA1 prevents these BNIP3 mutants from inducing mitochondrial damage and cell death [16]. Additionally, this supports a model in which BNIP3-induced mitochondrial damage and cell death depend on the C-terminal BNIP3-OPA1 interaction, and BNIP3-induced autophagy depends on the N-terminal BNIP3-LC3-II interaction (Fig 6) [7, 11, 12, 16].

BNIP3 is uniquely positioned such that it can regulate both pro-survival and pro-death activities. It was previously reported that phosphomimetic mutations of two serine residues (S17 and S24) flanking the LIR sequence near the BNIP3 N-terminus promotes BNIP3-induced autophagy [12]. Importantly, the authors did not report any effects on mitochondrial damage or cell death [12]. Here we have shown that phosphorylation of the BNIP3 C-terminus inhibits BNIP3-induced cell death while allowing autophagy to proceed (Figs 4 and 5). Thus, phosphorylation acts as a switch between the pro-survival (autophagy) and pro-death (cytotoxic) activities of the protein. Therefore, the net result of BNIP3 expression in any given cell will depend upon the expression and activation status of kinases and phosphatases that regulate BNIP3 phosphorylation. Factors that might alter kinase activity include cAMP or cGMP levels, calcium availability, and growth factors, such as EGF or IGF, which have been shown to alter BNIP3 function [31, 35]. For this reason, it is not surprising that the literature is often contradictory in ascribing pro-survival and pro-death roles to BNIP3 in different cell types and microenvironments. This is particularly the case in oncology, where it is observed that in some cancers, such as pancreatic, gastric, and colorectal cancers, BNIP3 expression is suppressed or silenced by DNA methylation [54, 55], suggesting that the cells have blocked BNIP3 expression to evade cell death. In other cases, including endometrial, breast, and lung cancers, high BNIP3 expression is associated with poorer prognosis and a more aggressive phenotype [33, 56–58].

The fact that some tumors tolerate BNIP3 expression suggests that these cells block the pro-death functions of BNIP3. Our observation that C-terminal BNIP3 phosphorylation increases in both lung carcinoma and breast cancer cells during nutrient deprivation and hypoxia suggests that the cells might use C-terminal phosphorylation to block BNIP3 cytotoxicity (Fig 7E and 7F). This is consistent with evidence that endometrial, breast, and lung cancers have increased kinase activity relative to healthy tissue [59–61]. Similarly, some types of breast cancer also have increased PKA and PKC activity, and evidence suggests that this increased PKA activity contributes to the resistance of Her2-positive breast cancers to trastuzumab antibody therapy [60, 62]. In these examples, the increased PKA activity in solid tumors, which often tolerate nutrient deprivation and hypoxia, could protect BNIP3-expressing cancer cells from cell death in part by increasing phosphorylation of the BNIP3 C-terminus. These observations also suggest that BNIP3 phosphorylation could represent a new therapeutic target in transformed cells, where decreasing C-terminal BNIP3 phosphorylation could increase BNIP3-dependent activation of cell death.

## Methods

### Materials

Bafilomycin A1, Rapamycin, and 8-Bromoadenosine-3',5'-cyclic monophosphate sodium salt (8-Br-cAMP) were purchased from EMD Millipore (Darmstadt, Germany); 3-isobutyl-1-methylxanthine (IMBX) was purchased from Cayman Chemical (Ann Arbor, MI). Fluorescent probes Mitotracker Green FM, Lysotracker Red DND-99, Dihydroethidium (DHE), and MitoSOX Red mitochondrial superoxide indicator were purchased from Molecular Probes (Grand Island, NY). JC1 and DCF were purchased from Sigma (St Louis, MO), and Annexin V was purchased from BD Biosciences (San Jose, CA). Antibodies were obtained as follows: mouse monoclonal anti-BNIP3 (Sigma, St Louis, MO, B7931), mouse monoclonal anti-His-tag (Qiagen, Valencia, CA, 34650), rabbit polyclonal anti-VDAC1 (Novus Biologicals, Littleton, CO, NB 100–695), rabbit monoclonal anti-PKA substrate (Cell Signaling Technology, Danvers, MA, 9624S), rabbit polyclonal anti-LC3 (Novus Biologicals, Littleton, CO, NB 100–2220), mouse monoclonal anti-SQSTM1 (Abcam, Cambridge, MA, ab56416), mouse monoclonal anti-OPA1 (BD Biosciences, 612606), rabbit polyclonal anti-BCL2 (Santa Cruz Biotechnology, Inc., Dallas, TX, sc-492), mouse monoclonal anti-MT-CO2 (Abcam, ab3298), rabbit monoclonal anti-MT-CO2 (Abcam, ab79393), rabbit polyclonal anti-ACTB (actin, beta) (Cell Signaling Technology, Danvers, MA, 4968), and mouse monoclonal anti-GAPDH (Acris, San Diego, CA, ACR001PT).

### Cloning

*BNIP3* DNA (provided by Dr. Gerald Dorn, Washington University School of Medicine) was inserted into pQE30 to generate N-terminal His-tagged BNIP3. This construct was transferred to the pTre-Tight vector (Clontech Laboratories, Inc., Mountain View, CA) for expression in mammalian cells, and the WT sequence mutated via PCR using the following mutagenesis primers: ΔR (5’-GGA TCT ATA TTG GAT AGC GTC TGA CAA CCT CC-3’ and 5’-GGT GCT GGT GGA GGT TGT CAG ACG CTA TCC AAT ATA G-3’), ΔTM (5’-GGC ATA TTC TCT GCA GAA TTT CTG AAA TAG TGA CTT CCA TCT CTG CTG CTC-3’ and 5’- GAG AGA GCA GCA GAG ATG GAA GTC ACT ATT TCA GAA ATT CTG CAG AG-3’), T188A (5’-TTG GAA GGC GTC TGG CAA CCT CCA CC-3’ and 5’-GCT GGT GGA GGT TGC CAG ACG CCT T-3’), T188D (5’-TTG GAA GGC GTC TGG ATA CCT CCA CC-3’ and 5’-GGT GCT GGT GGA GGT ATC CAG ACG C-3’), 6N (5’-GGA AGG CGT CTG AAC AAC AAC AAC AAC AAC TTT TGA AAG CTT GTC G-3’ and 5’-CGT CGA CAA GCT TTC AAA AGT TGT TGT TGT TGT TGT TCA GAC GCC-3’), and 6D (5’-GGA AGG CGT CTG GAC GAC GAC GAC GAC GAC TTT TGA AAG CTT GTC G-3’ and 5’-CGT CGA CAA GCT TTC AAA AGT CGT CGT CGT CGT CGT CCA GAC GCC-3’). Each mutant was sequenced to confirm mutagenesis. Upon generating 6N BNIP3, the six S/T residues were replaced with asparagine instead of alanine to preserve the hydrophilic and hydrogen bonding nature of the C-terminus. The WT *BNIP3*-*dsRed* monomer C1 vector (Clontech, 632466), was provided by Dr. Abhinav Diwan (Washington University School of Medicine) [8], and site-directed mutagenesis was used to generate the same BNIP3 mutants as described above. The pcDNA3 vector containing *BCL2* was also obtained from Addgene (plasmid 8768, provided by Stanley Korsmeyer) [63].

### Cell Culture

HEK 293 Tet On cells (purchased from Clontech Laboratories, Inc., Cat # 631182) were maintained in α-MEM with 10% Tet system approved FBS (Clontech Laboratories, Inc.) and 100 μg/mL G418. Double stable HEK 293 Tet On cells expressing BNIP3 (WT or mutant) were selected by the addition of 15 μg/mL hygromycin to the previously described media, and BNIP3 expression was induced with doxycycline for 48 hr unless otherwise noted. Transfection was performed using Lipofectamine 2000 (Life Technologies), according to manufacturer recommendations. 293T, A549, AU565, and MDA-MB-231 cells were purchased from the ATCC (Manassas, VA) and maintained per ATCC guidelines. To monitor autophagy, GFP-LC3 (provided by A. Diwan) was transiently transfected in double stable HEK 293 Tet On-BNIP3 cells. As a positive control, HEK 293 Tet On cells were treated with 100 nM Rapamycin for 18 hr to activate autophagy. Autophagosome clearance was inhibited using 50 nM Bafilomycin A1 for 12 hr. A time course of autophagy activation was performed using GFP-LC3 by fixing cells after BNIP3 expression for 24, 48, or 72 hr.

Intracellular cAMP levels were elevated by treating cells with 1 mM 8-Br-cAMP and 100 μM IBMX. Cellular stress was induced by treating HEK 293 cells with 150 μM H_2_O_2_ for 120 min, or by treating cells with 10 μM FCCP for 120 min. Nutrient deprivation was achieved by replacing cell culture media with Hank’s Balanced Salt Solution (Life Technologies), and cells were subjected to hypoxia using a modular incubator chamber (Billups-Rothenberg, Inc.) containing 5% CO_2_ and 95% N_2_ for up to 48 hr.

### Confocal Microscopy

HEK 293 Tet On cells expressing each form of BNIP3 were grown on glass coverslips coated with 50 mM poly-l-lysine (Sigma) for 24 hr. To examine GFP-LC3 puncta and BNIP3-OPA1 colocalization, cells were fixed using 4% electron microscopy grade paraformaldehyde (EMS) for 10 min, quenched for 5 min using quenching buffer (50 mM Tris Cl, pH 7.5, 100 mM NaCl) and nuclei stained with 0.5 μg/mL DAPI (Sigma). Coverslips were mounted using Prolong Gold (Molecular Probes, P36930). Colocalization analysis of OPA1 and BNIP3 with mitochondria was achieved by probing cells with 300 nM Mitotracker CMXRos for 1 hr prior to fixation. Live cell imaging using 2.5 μM JC1 or 1 nM Lysotracker Red was achieved by mounting stained cells in Minimum Essential Medium, no phenol red, with 10% FBS. High resolution images were collected using a Zeiss LSM 700, and quantification of fluorescence was performed using ImageJ (NIH) [64]. In each case, autothresholding was applied prior to analysis using the find maxima or analyze particles functions. Colocalization of BNIP3 or OPA1 with either Mitotracker CMXRos or MT-CO2, as well as BNIP3-OPA1 colocalization, was quantified using Zen software (Zeiss).

### Transmission Electron Microscopy

HEK 293 cell pellets were fixed in a modified Karnovsky's fixative buffer (3% glutaraldehyde, 1% paraformaldehyde in 0.1 M sodium cacodylate), post-fixed in 2% osmium tetroxide in 0.1 M sodium cacodylate buffer for 1 hr., en bloc stained with 2% aqueous uranyl acetate for 30 min, dehydrated in graded acetone, and embedded in PolyBed 812 (Polysciences, 08792–1). Cell pellet blocks were sectioned at 90 nm thick, post stained with Venable's lead citrate, and viewed with a JEOL model 1400EX electron microscope. Digital images were acquired using the Gatan Orius high definition CCD, 11 megapixel TEM camera. Quantification of mitochondrial elongation was performed independently by 5 individuals, and mitochondrial area was measured using ImageJ (NIH).

### Flow Cytometry

Flow cytometry was performed using an Accuri C6 flow cytometer. Fluorescent probes were used as follows: 100 nM Mitotracker Green FM, 30 min; 2.5 μM JC1, 30 min; 10 μM DHE, 10 min; 500 nM DCF, 10 min; 5 μM MitoSox, 15 min; 50 nM Lyostracker Red, 30 min; 20 nM DiOC6, 15 min; Annexin V, per manufacturer recommendations. Controls for mitochondrial membrane potential assays were employed using treatment of control cells with 5 μg/mL Oligomycin A (Oligo A) and 10 μM FCCP for 30 min. A minimum of 30,000 events were collected for each sample, and compensation was performed for JC1 dual color fluorescence. Data analysis was performed using Accuri C6 software. JC1 fluorescence was quantified using the ratio of red:green JC1 fluorescence, which represents the ratio of polarized:depolarized mitochondria.

### Mitochondrial Fractionation and Alkaline Extraction

Subcellular fractionation was performed using the protocol previously described, with minor changes [65]. Briefly, cells were lysed in fractionation buffer (250 mM sucrose, 20 mM HEPES, pH7.4, 10 mM KCl, 1.5 mM MgCl_2_, 1 mM EDTA, 1 mM EGTA, 1 mM DTT, cOmplete protease inhibitor (Roche, Basel, Switzerland), and 50 mM β-glycerophosphate). Following centrifugation at 720g, the supernatant was centrifuged at 10,000g, and the mitochondrial pellet washed with fractionation buffer. Samples were analyzed by Western blot. Alkaline extraction of mitochondria was performed as previously described [65]. Briefly, isolated mitochondria were suspended in 100 mM Na_2_CO_3_, pH 11.3, incubated on ice 20 min, and centrifuged at 10,000g for 15 min. Supernatant and pellet were solubilized in 2x Laemmli buffer and analyzed via Western blot.

### Quantitative PCR

Genomic DNA was purified from HEK 293 cells expressing each form of BNIP3 using a DNeasy Blood and Tissue Kit (Qiagen). Mitochondrial DNA (mtDNA) was quantified by qPCR, using primers specific for ND1 (mitochondrially-encoded NADH dehydrogenase, subunit 1) 5’- CCCTAAAACCCGCCACATCT-3’ and 5’-GAGCGATGGTGAGAGCTAAGGT-3’ and a reference gene B2M (beta-2-microglobulin) 5’-TGCTGTCTCCATGTTTGATGTATCT-3’ and 5’-TCTCTGCTCCCCACCTCTAAGT-3’, as previously described [66, 67]. Quantitative PCR was achieved using a ViiA 7 Real Time PCR System (Applied Biosystems) with SYBR Select Master Mix (Life Technologies). Melt curves were monitored to ensure specificity of the primers. Relative quantification of mtDNA were calculated as 2^ΔΔCt^, where ΔΔCt represents the difference between ΔCt _control_ (no BNIP3) and ΔCt _sample_ (each form of BNIP3) and ΔCt represents the difference Ct _target_ (ND1) and Ct _endogenous control_ (B2M).

### Co-immunoprecipitation

Co-immunoprecipitation assays were performed as previously described [16]. Cells were lysed using lysis buffer (50 mM Tris-Cl, pH 7.5, 250 mM NaCl, 5mM EDTA, 5 mM EGTA, 1% Triton X-100, cOmplete protease inhibitor) and applied to antibody-coated Sheep anti-Mouse IgG Dynabeads (Life Technologies) for 4 hr at 4°C. The samples were washed 3 times with washing buffer (50 mM Tris-Cl, pH 7.5, 250 mM NaCl, 5mM EDTA, 5 mM EGTA, 0.05% Triton X-100, cOmplete protease inhibitor), eluted using 2x Laemmli buffer, and analyzed by Western blot.

### SDS PAGE and Western Blotting

Protein extracts, prepared in 2x Laemmli buffer, were subjected to SDS PAGE using Mini-PROTEAN TGX gradient (5–15%) gels (Bio-Rad, Hercules, CA) and transferred to PVDF membrane for 1 hr. Lysates treated without or with BAF, to monitor conversion of LC3-I to LC3-II, were separated on 16.5% Mini-PROTEAN Tris-Tricine gels for accurate detection of both LC3 species. Membranes were blocked in SuperBlock blocking buffer in TBS (Pierce, Rockford, IL). Following application of primary antibody (1:1000 dilution), either donkey anti-rabbit (Pierce) or goat anti-mouse (Pierce) IgG HRP conjugated secondary antibody was applied, and blots were developed using SuperSignal West Pico Chemiluminescent Substrate (Pierce). Densitometry was performed using ImageJ (NIH) [64].

### Mass Spectrometry

His-tagged WT BNIP3 was purified from HEK 293 Tet On cells under denaturing conditions (8M urea). Following denaturation, the cellular lysate was passed through a Ni-NTA column and washed extensively. Protein was eluted from the column using elution buffer (50 mM NaH_2_PO_4_, 300 mM NaCl, 8 M urea, 1 M imidazole, pH 6.5) and the eluate reduced and alkylated. Peptides were generated using trypsin digestion, and the sample analyzed by LC-MS/MS using a linear quadrupole ion-trap and an Orbitrap (LTQ-Orbitrap XL, Thermo Fisher Scientific, Waltham, MA) as previously described [68].

### Statistical Analysis

Statistical analysis was performed using Graph Pad (version 4.02). In each experiment, a one-way ANOVA was performed, followed by a Dunnett test to compare cells expressing WT or each BNIP3 mutant to control cells lacking BNIP3. In each comparison, p < 0.05 was considered significant. In each figure, significant differences between control cells (without BNIP3) and cells expressing each BNIP3 mutant are denoted by * p<0.05, ** p<0.01, and *** p<0.001. Significant differences between HEK 293 cells expressing WT BNIP3 and either control cells (without BNIP3) or cells expressing each BNIP3 mutant are denoted by #p<0.05, ##p<0.01, ###p<0.001. Post-hoc Tukey tests were performed for statistical analysis of paired samples, to allow for the comparison of the complementary phosphomimetic and nonphosphorylated BNIP3 mutants (ΔTM vs ΔR BNIP3, T188D vs T188A BNIP3, and 6D vs 6N BNIP3). In each figure, significant differences between each complementary pair of nonphosphorylated and phosphomimetic BNIP3 mutants are denoted by brackets. When applicable, additional statistical analysis between treatment conditions is noted in figure captions. Error bars represent SEM.

## Supporting Information

S1 Fig Phosphomimetic mutation of the BNIP3 C-terminus prevents BNIP3-induced loss of mtDNA content. Mitochondrial DNA content was quantified by PCR, using primers specific for ND1 (mitochondrially-encoded NADH dehydrogenase, subunit 1) and a reference gene B2M (beta-2-microglobulin). Results are expressed as the relative quantification (RQ), calculated as 2^ΔΔCt^, where ΔΔCt represents the difference between ΔCt _control_ (no BNIP3) and ΔCt _sample_ (each form of BNIP3). The bar graph represents the average RQ from 4 independent experiments. Significant differences between control cells (without BNIP3) and cells expressing each BNIP3 mutant are denoted by * p<0.05, ** p<0.01, and *** p<0.001. Significant differences between HEK 293 cells expressing WT BNIP3 and either control cells or cells expressing each BNIP3 mutant are denoted by #p<0.05, ##p<0.01, ###p<0.001; significant differences between pairs of complementary BNIP3 mutants are denoted in brackets.
(TIF)

S2 Fig Phosphomimetic BNIP3 does not reduce the mitochondrial membrane potential of HEK 293 cells. A) Quantification of red JC1 puncta, observed by confocal microscopy. Control cells were treated with Oligomycin A1 (Oligo A) or FCCP to hyperpolarize or depolarize mitochondria, respectively. Bar graph represents results from 3 independent experiments in which a minimum of 30 cells were observed. B) HEK 293 cells expressing each form of BNIP3 were probed with DiOC6, and the fluorescence intensity measured by flow cytometry analysis of a minimum of 30,000 events per sample. Results represent the mean fluorescence intensity, calculated from 3 independent experiments. Control cells were treated with Oligomycin A1 (Oligo A) or FCCP to hyperpolarize or depolarize mitochondria, respectively. Significant differences between control cells (without BNIP3) and cells expressing each BNIP3 mutant are denoted by * p<0.05, ** p<0.01, and *** p<0.001; significant differences between cells expressing WT BNIP3 and either control cells or cells expressing each BNIP3 mutant are denoted by # p<0.05, ## p<0.01, and ### p<0.001; significant differences between complementary pairs of mutants are denoted in brackets.
(TIF)

S3 Fig C-terminal BNIP3 phosphorylation prevents BNIP3-induced increases in ROS. Representative flow cytometry histograms of HEK 293 cells expressing WT or phosphomutant BNIP3, probed with (A) DHE, (B) DCF-DA, and (C) MitoSox to quantify ROS. For each fluorescent probe, two histograms are provided to demonstrate the relative fluorescence intensities of HEK 293 cells expressing either ΔTM, WT, T188A, or T188D BNIP3 (left histogram) or either ΔTM, WT, 6N, or 6D BNIP3 (right histogram); to allow for comparison between histograms, each pair of histograms contains the same ΔTM and WT BNIP3 examples. The red vertical line denotes the mean fluorescence intensity of HEK 293 cells expressing ΔTM BNIP3. Bar graphs displaying the full quantification of this data are provided in Fig 3C–3E.
(TIF)

S4 Fig Phosphorylation of the BNIP3 C-terminus does not prevent the activation of autophagy. A) HEK 293 cells expressing each form of BNIP3 were treated with or without BAF, and LC3 was detected by Western blot. To quantify autophagic flux, the ratio of LC3-II/ACTB between BAF treated cells and untreated cells was calculated. B) Ratio of SQSTM1/ACTB between BAF treated and untreated cells expressing each form of BNIP3, calculated following the same treatment as described in (A). C) The time course of autophagy activation, measured by the number of GFP-LC3 puncta per cell in HEK 293 cells expressing each BNIP3 mutant for 24, 48, or 72 hr, is similar in cells expressing nonphosphorylated or phosphomimetic BNIP3. In each condition, a minimum of 30 cells were observed in 3 independent experiments. Significant differences between control cells (without BNIP3) and cells expressing each BNIP3 mutant for 24 hr are denoted by * p<0.05, ** p<0.01, and *** p<0.001; significant differences between control cells and cells expressing each BNIP3 mutant for 48 hr are denoted by # p<0.05, ## p<0.01, and ### p<0.001; significant differences between control cells and cells expressing each BNIP3 mutant for 72 hr are denoted by $ p<0.05, $ $ p<0.01, and $ $ $ p<0.001.
(TIF)

S5 Fig Phosphorylation of the BNIP3 C-terminus does not alter the BCL2-BNIP3 protein-protein interaction. Detection of BCL2 by Western blot following immunoprecipitation of WT or mutant BNIP3 using an α-HIS tag antibody. WCL = whole cell lysate.
(TIF)

S6 Fig OPA1 remains localized to mitochondria following expression of WT or phosphomutant BNIP3. (A) Endogenous and exogenous OPA1 expression levels in HEK 293 cells expressing each form of BNIP3. To account for the reduced level of endogenous OPA1 in cells expressing WT or nonphosphorylated BNIP3, co-immunoprecipitation assays and colocalization analysis were performed following transient exogenous OPA1 expression. (B) Western blot analysis of endogenous OPA1 subcellular localization in cells expressing each form of BNIP3, showing cytosolic and mitochondrial pellet fractions. (C) Subcellular localization of OPA1 in HEK 293 cells expressing each form of BNIP3 following transient transfection of OPA1. Total OPA1 was detected using a monoclonal α-OPA1 antibody, and Mitotracker CMXRos was used to detect mitochondria and monitor OPA1 localization at mitochondria by confocal miscroscopy. Scale bar represents 10 μm. (D) Mitochondrial localization of BNIP3, detected using Mitotracker CMXRos and an α-His antibody specific to His-tagged WT or mutant BNIP3. Scale bar represents 10 μm. (E) Quantification of OPA1-Mitotracker CMXRos colocalization in HEK 293 cells expressing each form of BNIP3. (F) Quantification of BNIP3 localization to mitochondria, represented by the number of colocalized Mitotracker CMXRos-BNIP3 pixels per cell. For each bar graph, significant differences in colocalization between control cells (without BNIP3) and cells expressing each BNIP3 mutant are denoted by * p<0.05, ** p<0.01, and *** p<0.001; significant differences between cells expressing WT BNIP3 and either control cells (no BNIP3) or cells expressing each BNIP3 mutant are denoted by # p<0.05, ## p<0.01, and ### p<0.001.
(TIF)

S7 Fig Phosphorylation of the BNIP3 C-terminus reduces the colocalization of BNIP3 with OPA1. (A) Analysis of mitochondrial localization of BNIP3 and OPA1 by confocal microscopy. BNIP3 (dsRed tagged) and OPA1 (α-OPA1, FITC secondary) both localize to mitochondria, visualized by MT-CO2 (α-MT-CO2, CF 405M secondary). A minimum of 30 cells expressing each form of BNIP3 were examined in 3 independent experiments. Scale bar represents 10 μm. Traces of fluorescence intensity provide examples of the colocalization of MT-CO2, OPA1, and BNIP3 in HEK 293 cells expressing each form of BNIP3. (B) Quantification of OPA1 localization to mitochondria, calculated as the percent of OPA1 pixels colocalized with MT-CO2 per cell. (C) Quantification of BNIP3 localization to mitochondria, calculated as the percent of BNIP3 pixels colocalized with MT-CO2 per cell. (D) Quantification of BNIP3-OPA1 colocalization in cells probed with dsRed BNIP3, FITC-labeled OPA1, and CF-labeled MT-CO2, calculated as the percent of OPA1 pixels colocalized with BNIP3. For each bar graph, significant differences in colocalization between control cells (without BNIP3) and cells expressing each BNIP3 mutant are denoted by * p<0.05, ** p<0.01, and *** p<0.001; significant differences between cells expressing WT BNIP3 and either control cells or cells expressing each BNIP3 mutant are denoted by # p<0.05, ## p<0.01, and ### p<0.001.
(TIF)

S8 Fig Uncropped Western blots for Figs 1–7 and S5 and S6 Figs. (TIF)
