# Supplementary figures and images for: Phosphorylation of the BNIP3 C-Terminus Inhibits Mitochondrial Damage and Cell Death without Blocking Autophagy
Source: PLoS One. 2015 Jun 23;10(6):e0129667. doi: 10.1371/journal.pone.0129667 (PMC4477977; doi:10.1371/journal.pone.0129667)

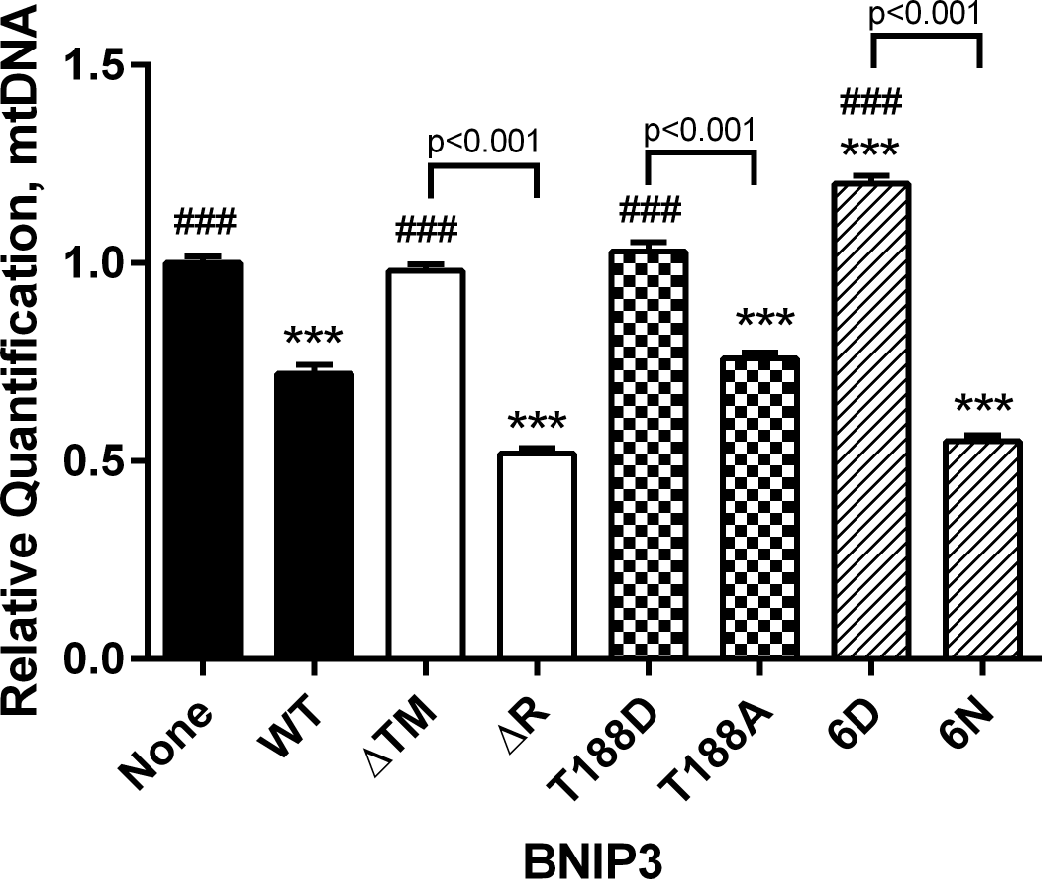

Supplement: S1 Fig — Mitochondrial DNA content was quantified by PCR, using primers specific for ND1 (mitochondrially-encoded NADH dehydrogenase, subunit 1) and a reference gene B2M (beta-2-microglobulin). Results are expressed as the relative quantification (RQ), calculated as 2ΔΔCt, where ΔΔCt represents the difference between ΔCt control (no BNIP3) and ΔCt sample (each form of BNIP3). The bar graph represents the average RQ from 4 independent experiments. Significant differences between control cells (without BNIP3) and cells expressing each BNIP3 mutant are denoted by * p<0.05, ** p<0.01, and *** p<0.001. Significant differences between HEK 293 cells expressing WT BNIP3 and either control cells or cells expressing each BNIP3 mutant are denoted by #p<0.05, ##p<0.01, ###p<0.001; significant differences between pairs of complementary BNIP3 mutants are denoted in brackets. (TIF) [file pone.0129667.s001.tif]

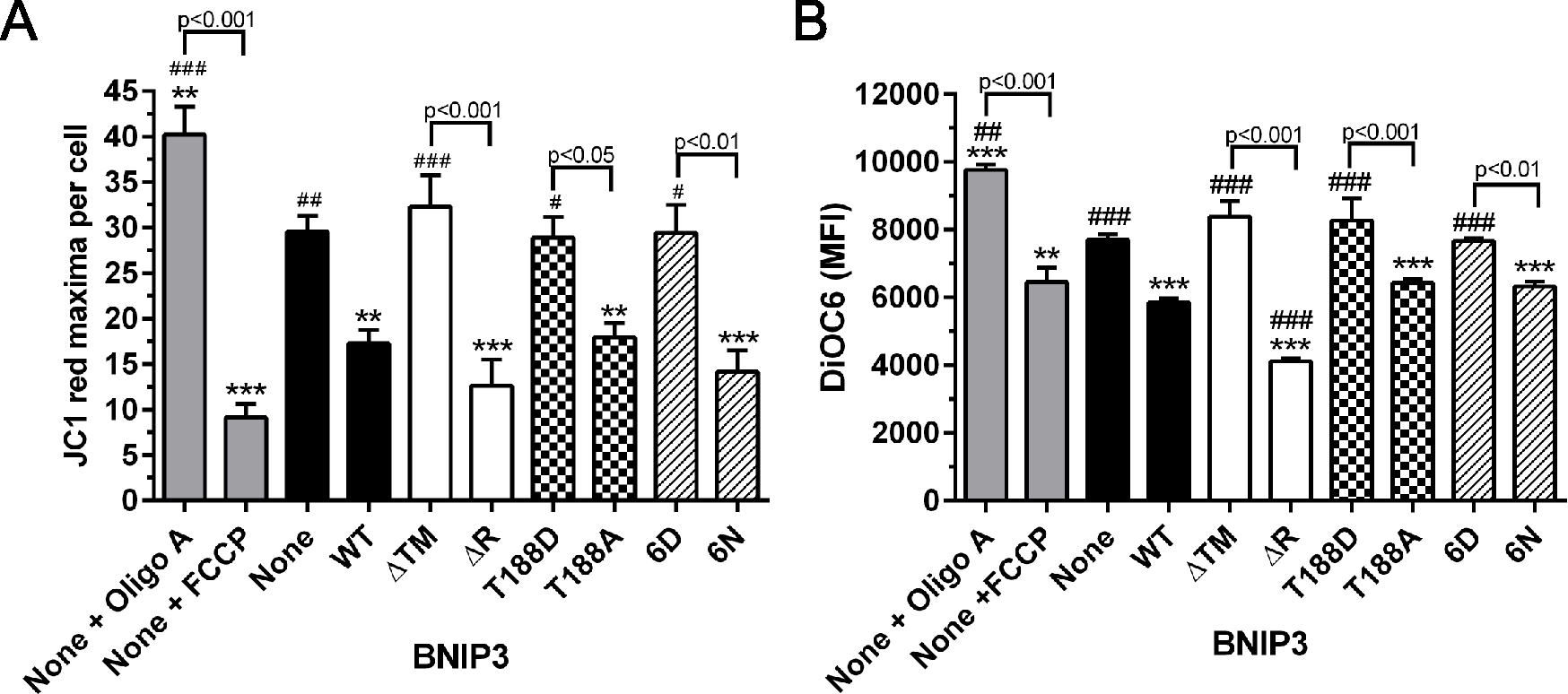

Supplement: S2 Fig — A) Quantification of red JC1 puncta, observed by confocal microscopy. Control cells were treated with Oligomycin A1 (Oligo A) or FCCP to hyperpolarize or depolarize mitochondria, respectively. Bar graph represents results from 3 independent experiments in which a minimum of 30 cells were observed. B) HEK 293 cells expressing each form of BNIP3 were probed with DiOC6, and the fluorescence intensity measured by flow cytometry analysis of a minimum of 30,000 events per sample. Results represent the mean fluorescence intensity, calculated from 3 independent experiments. Control cells were treated with Oligomycin A1 (Oligo A) or FCCP to hyperpolarize or depolarize mitochondria, respectively. Significant differences between control cells (without BNIP3) and cells expressing each BNIP3 mutant are denoted by * p<0.05, ** p<0.01, and *** p<0.001; significant differences between cells expressing WT BNIP3 and either control cells or cells expressing each BNIP3 mutant are denoted by # p<0.05, ## p<0.01, and ### p<0.001; significant differences between complementary pairs of mutants are denoted in brackets. (TIF) [file pone.0129667.s002.tif]

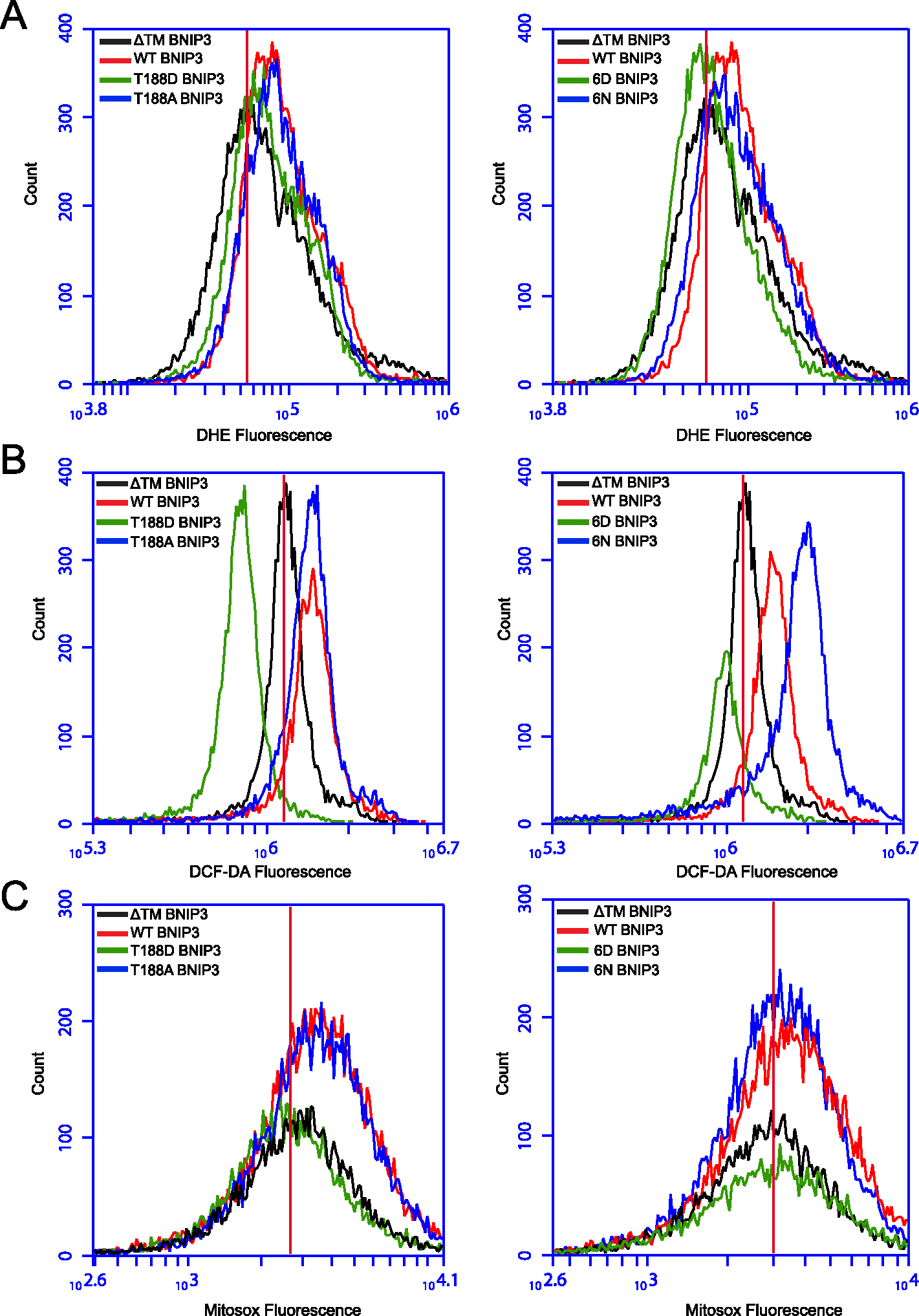

Supplement: S3 Fig — Representative flow cytometry histograms of HEK 293 cells expressing WT or phosphomutant BNIP3, probed with (A) DHE, (B) DCF-DA, and (C) MitoSox to quantify ROS. For each fluorescent probe, two histograms are provided to demonstrate the relative fluorescence intensities of HEK 293 cells expressing either ΔTM, WT, T188A, or T188D BNIP3 (left histogram) or either ΔTM, WT, 6N, or 6D BNIP3 (right histogram); to allow for comparison between histograms, each pair of histograms contains the same ΔTM and WT BNIP3 examples. The red vertical line denotes the mean fluorescence intensity of HEK 293 cells expressing ΔTM BNIP3. Bar graphs displaying the full quantification of this data are provided in Fig 3C–3E. (TIF) [file pone.0129667.s003.tif]

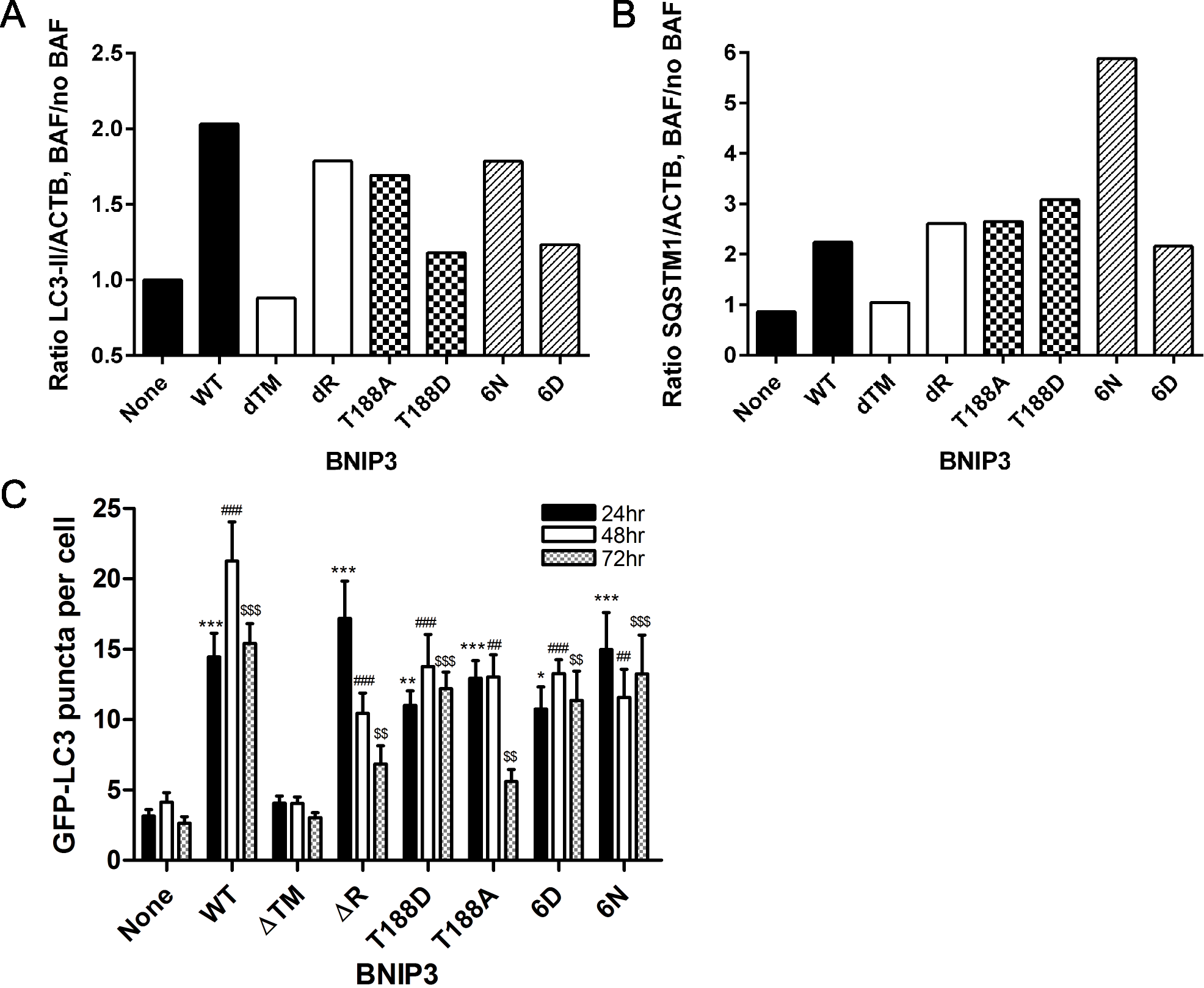

Supplement: S4 Fig — A) HEK 293 cells expressing each form of BNIP3 were treated with or without BAF, and LC3 was detected by Western blot. To quantify autophagic flux, the ratio of LC3-II/ACTB between BAF treated cells and untreated cells was calculated. B) Ratio of SQSTM1/ACTB between BAF treated and untreated cells expressing each form of BNIP3, calculated following the same treatment as described in (A). C) The time course of autophagy activation, measured by the number of GFP-LC3 puncta per cell in HEK 293 cells expressing each BNIP3 mutant for 24, 48, or 72 hr, is similar in cells expressing nonphosphorylated or phosphomimetic BNIP3. In each condition, a minimum of 30 cells were observed in 3 independent experiments. Significant differences between control cells (without BNIP3) and cells expressing each BNIP3 mutant for 24 hr are denoted by * p<0.05, ** p<0.01, and *** p<0.001; significant differences between control cells and cells expressing each BNIP3 mutant for 48 hr are denoted by # p<0.05, ## p<0.01, and ### p<0.001; significant differences between control cells and cells expressing each BNIP3 mutant for 72 hr are denoted by $ p<0.05, $ $ p<0.01, and $ $ $ p<0.001. (TIF) [file pone.0129667.s004.tif]

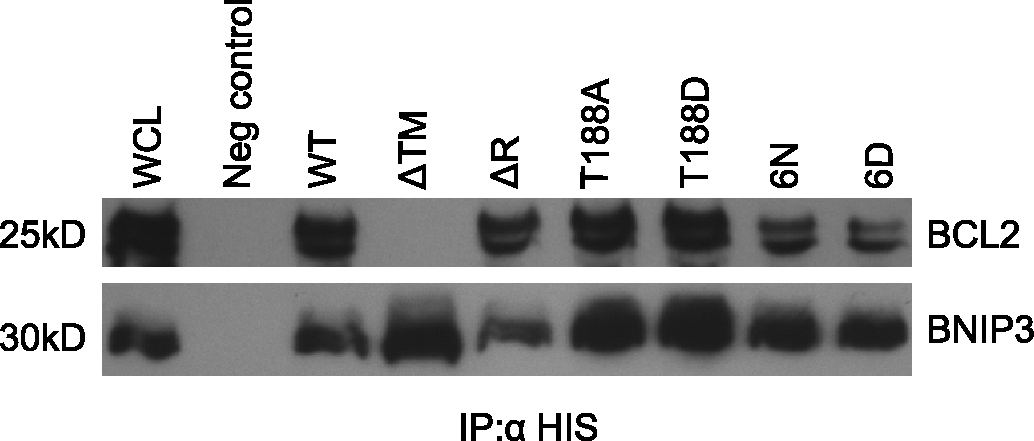

Supplement: S5 Fig — Detection of BCL2 by Western blot following immunoprecipitation of WT or mutant BNIP3 using an α-HIS tag antibody. WCL = whole cell lysate. (TIF) [file pone.0129667.s005.tif]

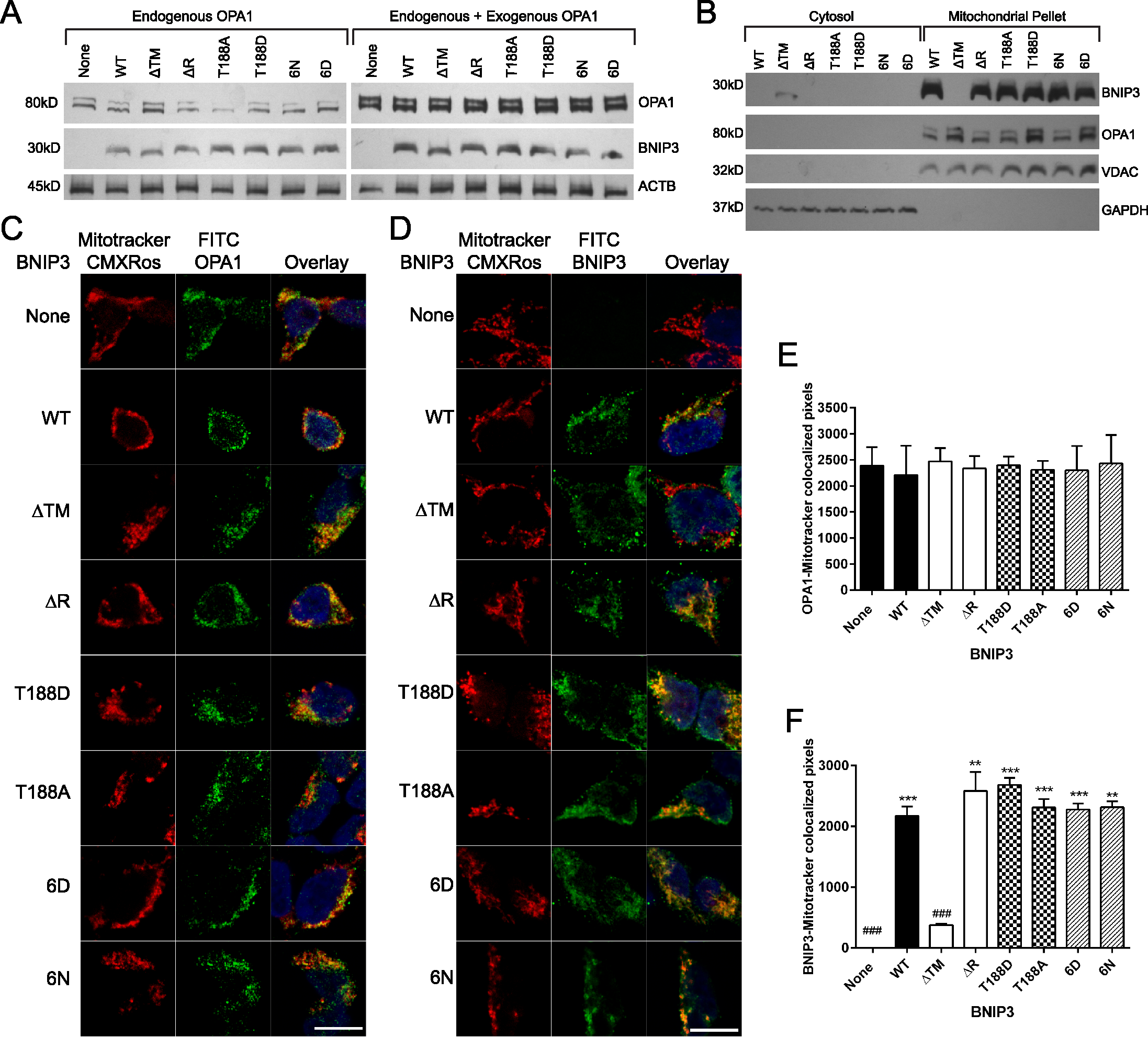

Supplement: S6 Fig — (A) Endogenous and exogenous OPA1 expression levels in HEK 293 cells expressing each form of BNIP3. To account for the reduced level of endogenous OPA1 in cells expressing WT or nonphosphorylated BNIP3, co-immunoprecipitation assays and colocalization analysis were performed following transient exogenous OPA1 expression. (B) Western blot analysis of endogenous OPA1 subcellular localization in cells expressing each form of BNIP3, showing cytosolic and mitochondrial pellet fractions. (C) Subcellular localization of OPA1 in HEK 293 cells expressing each form of BNIP3 following transient transfection of OPA1. Total OPA1 was detected using a monoclonal α-OPA1 antibody, and Mitotracker CMXRos was used to detect mitochondria and monitor OPA1 localization at mitochondria by confocal miscroscopy. Scale bar represents 10 μm. (D) Mitochondrial localization of BNIP3, detected using Mitotracker CMXRos and an α-His antibody specific to His-tagged WT or mutant BNIP3. Scale bar represents 10 μm. (E) Quantification of OPA1-Mitotracker CMXRos colocalization in HEK 293 cells expressing each form of BNIP3. (F) Quantification of BNIP3 localization to mitochondria, represented by the number of colocalized Mitotracker CMXRos-BNIP3 pixels per cell. For each bar graph, significant differences in colocalization between control cells (without BNIP3) and cells expressing each BNIP3 mutant are denoted by * p<0.05, ** p<0.01, and *** p<0.001; significant differences between cells expressing WT BNIP3 and either control cells (no BNIP3) or cells expressing each BNIP3 mutant are denoted by # p<0.05, ## p<0.01, and ### p<0.001. (TIF) [file pone.0129667.s006.tif]

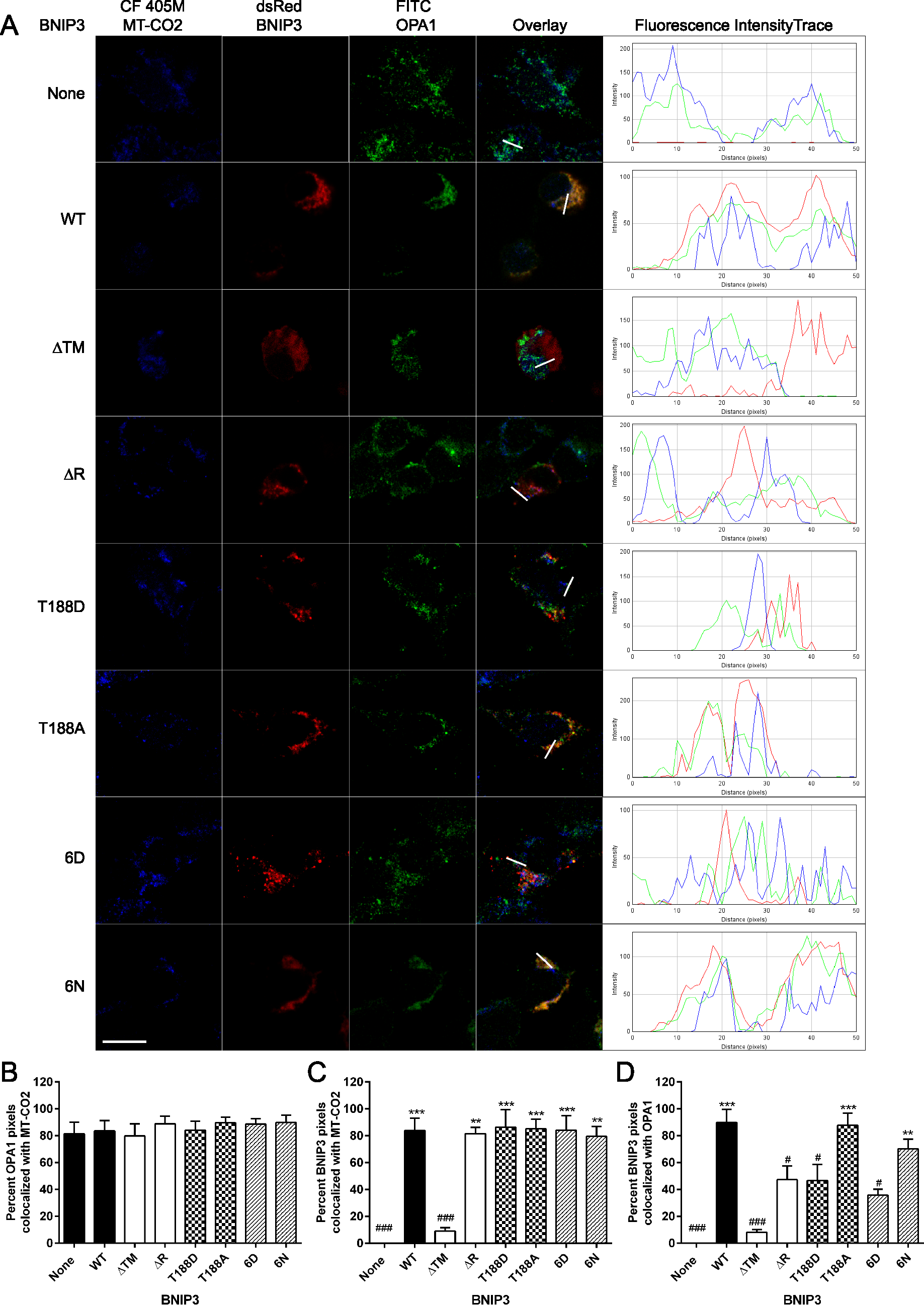

Supplement: S7 Fig — (A) Analysis of mitochondrial localization of BNIP3 and OPA1 by confocal microscopy. BNIP3 (dsRed tagged) and OPA1 (α-OPA1, FITC secondary) both localize to mitochondria, visualized by MT-CO2 (α-MT-CO2, CF 405M secondary). A minimum of 30 cells expressing each form of BNIP3 were examined in 3 independent experiments. Scale bar represents 10 μm. Traces of fluorescence intensity provide examples of the colocalization of MT-CO2, OPA1, and BNIP3 in HEK 293 cells expressing each form of BNIP3. (B) Quantification of OPA1 localization to mitochondria, calculated as the percent of OPA1 pixels colocalized with MT-CO2 per cell. (C) Quantification of BNIP3 localization to mitochondria, calculated as the percent of BNIP3 pixels colocalized with MT-CO2 per cell. (D) Quantification of BNIP3-OPA1 colocalization in cells probed with dsRed BNIP3, FITC-labeled OPA1, and CF-labeled MT-CO2, calculated as the percent of OPA1 pixels colocalized with BNIP3. For each bar graph, significant differences in colocalization between control cells (without BNIP3) and cells expressing each BNIP3 mutant are denoted by * p<0.05, ** p<0.01, and *** p<0.001; significant differences between cells expressing WT BNIP3 and either control cells or cells expressing each BNIP3 mutant are denoted by # p<0.05, ## p<0.01, and ### p<0.001. (TIF) [file pone.0129667.s007.tif]

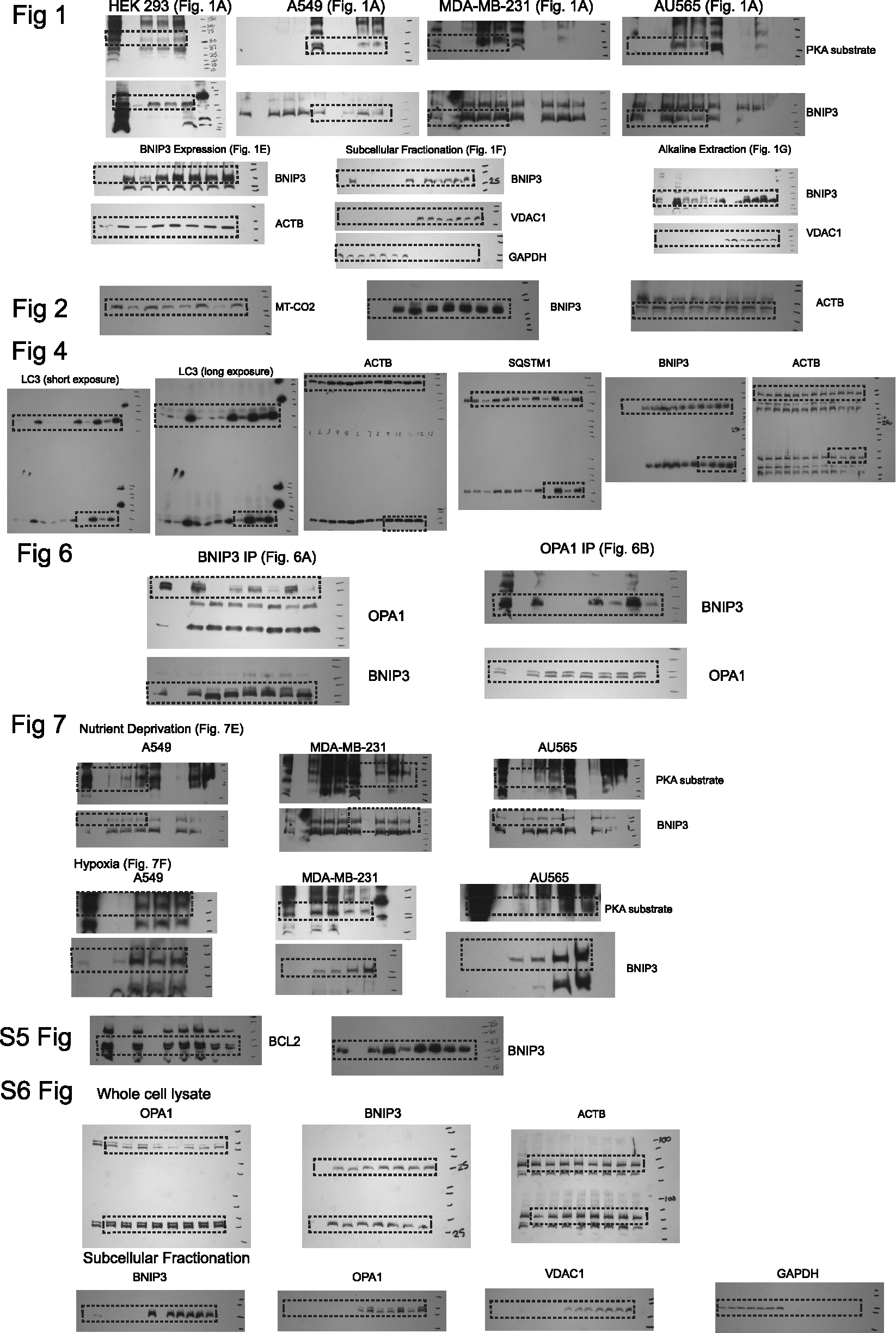

Supplement: S8 Fig — (TIF) [file pone.0129667.s008.tif]
